# Auxin-Inducible Degron System Reveals Temporal-Spatial Roles of HSF-1 and Its Transcriptional Program in Lifespan Assurance

**DOI:** 10.3389/fragi.2022.899744

**Published:** 2022-07-11

**Authors:** Allison C. Morphis, Stacey L. Edwards, Purevsuren Erdenebat, Lalit Kumar, Jian Li

**Affiliations:** Aging and Metabolism Research Program, Oklahoma Medical Research Foundation, Oklahoma, OK, United States

**Keywords:** aging, proteostasis, heat shock factor, auxin-inducible degron, temporal-spatial function, core chaperome, lifespan

## Abstract

HSF-1 is a key regulator of cellular proteotoxic stress response and is required for animal lifespan. In *C. elegans*, HSF-1 mediated heat shock response (HSR) declines sharply on the first day of adulthood, and HSF-1 was proposed to function primarily during larval stages for lifespan assurance based on studies using RNAi. The tissue requirement for HSF-1 in lifespan, however, is not well understood. Using the auxin-inducible degron (AID) system, we manage to uncouple the roles of HSF-1 in development and longevity. In wild-type animals, we find HSF-1 is required during the whole self-reproductive period for lifespan. This period is extended in long-lived animals that have arrested germline stem cells (GSC) or reduced insulin/IGF-1 signaling (IIS). While depletion of HSF-1 from any major somatic tissues during development results in severe defects, HSF-1 primarily functions in the intestine and likely neural system of adults to support lifespan. Finally, by combining AID and genome-wide transcriptional analyses, we find HSF-1 directly activates the transcription of constitutively-expressed chaperone and co-chaperone genes among others in early adulthood, which underlies its roles in longevity assurance.

## Introduction

HSF1 is best known as a key transcriptional activator of cellular heat shock response (HSR). Upon proteotoxic stress such as heat shock, HSF1 induces the expression of genes encoding molecular chaperones, detoxification enzymes, and protein degradation machinery to cope with stress-associated protein damage and misfolding (Vihervaara and Sistonen, 2014; Gomez-Pastor et al., 2018). It is well established that in *Drosophila* and mammalian cells, HSF1 activates the HSR by releasing paused RNA Polymerase II (Pol II) at promoter-proximal regions into productive elongation (Duarte et al., 2016; Mahat et al., 2016). Accumulating evidence suggests that HSF1 also has important roles in animal development, reproduction, and lifespan in both vertebrates and invertebrates (Li et al., 2017). These physiological functions of HSF1 are at least in part through promoting proteostasis, but HSF1’s transcriptional programs in these conditions are not identical to the HSR (Li et al., 2016).

Proteostasis decline is a primary hallmark of aging as accumulated protein misfolding and aggregation are observed in aged animals and underlie age-related diseases such as neurodegenerative disorders (Balch et al., 2008; López-Otín et al., 2013). Consistent with its role in maintaining proteostasis, HSF1 is a prominent lifespan and healthspan promoting factor in *C. elegans*, *Drosophila,* and mammals (Hsu et al., 2003; Morley and Morimoto, 2004; Pierce et al., 2010; Pierce et al., 2013; Merkling et al., 2015). Conversely, reduced HSF1 activities are observed in mouse models of Huntington’s and Parkinson’s diseases (Kim et al., 2016; Gomez-Pastor et al., 2017).

HSF-1, the *C. elegans* orthologue of HSF1, has well-established roles in lifespan assurance. RNAi-mediated knock-down or reduction-of-function mutant of HSF-1 significantly reduces lifespan and causes early onset of protein aggregation and physical declines (Hsu et al., 2003; Morley and Morimoto, 2004). On the contrary, over-expression of HSF-1 promotes longevity (Morley and Morimoto, 2004; Baird et al., 2014). In addition, longevity-promoting pathways including arrested germline stem cells (GSC) and reduced insulin/IGF-1 signaling (IIS) suppress proteotoxicity and extend lifespan in an HSF-1 dependent manner (Hansen et al., 2005; Cohen et al., 2006; Volovik et al., 2012; Shemesh et al., 2013). The contributions of HSF-1 in longevity have been attributed to its activities in stress response, as GSC arrest and low IIS enhance the HSR (Labbadia and Morimoto, 2015). However, HSF-1 is suggested to promote longevity beyond activating the HSR since over-expression of a C-terminally truncated HSF-1 that fails to robustly induce the HSR still extends *C. elegans* lifespan (Baird et al., 2014). However, the transcriptional program of endogenous HSF-1 and how HSF-1 regulates gene expression in the absence of external stress in adult somatic tissues are poorly understood.

To obtain deeper understanding of HSF-1’s functions in longevity, it is also important to precisely determine the temporal-spatial requirement for HSF-1 in lifespan. Previous work using RNAi shows that HSF1 activity is primarily required during larval stages for longevity (Volovik et al., 2012). This temporal profile is consistent with the traditional view that HSF-1 functions through the HSR since the HSR is under programmed repression at the onset of reproductive maturity on Day 1 of adulthood (Labbadia and Morimoto, 2015). However, as HSF-1 is essential for *C. elegans* larval development, it is difficult to uncouple the lifespan shortening effects with developmental defects. On the other hand, several studies have shown that over-expression of HSF-1 in specific somatic tissues such as neural cells is sufficient to extend lifespan (Morley and Morimoto, 2004; Douglas et al., 2015). However, the tissue-specific contributions of endogenous HSF-1 in longevity are not well characterized.

To better understand the temporal-spatial requirement for HSF-1 in lifespan assurance, in this study, we applied an auxin-inducible degron (AID) system to enable rapid depletion of HSF-1 post-larval development in a tissue-specific manner. We also combined HSF-1 depletion by AID with RNA-seq and ChIP-seq analyses to determine the transcriptional program of HSF-1 that underlies its roles in longevity.

## Results

### HSF-1 Predominantly Functions in Early Adulthood to Support Normal Lifespan

**FIGURE 1:**
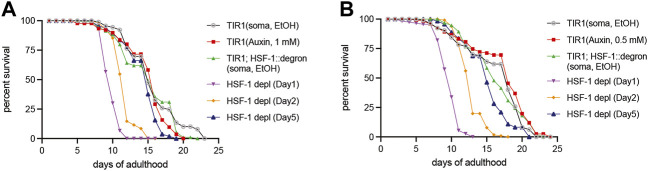
Temporal requirement for HSF-1 in lifespan. Lifespan analysis at 20°C upon pan-somatic depletion of HSF-1 by AID using 1 mM of auxin **(A)** or 0.5 mM of auxin **(B)**. The control strain, CA1200 (*eft-3p:tir1*) was mock treated with ethanol (EtOH) or treated with auxin from Day 1 of adulthood. The HSF-1 AID model, JTL611 (*eft-3p:tir1; hsf-1:degron*) were mock treated with ethanol (EtOH) from Day 1 of adulthood or transferred from EtOH to auxin plates at indicated time to initiate HSF-1 depletion.

HSF-1 is required for larval development as the null mutant exhibits larval arrest and lethality (Li et al., 2016; Morton and Lamitina, 2013). To examine HSF-1’s contribution to lifespan and uncouple it from its impacts on development, we took advantage of the AID system to deplete HSF-1 from the somatic tissues post-larval development. Our recent work has shown that expression of the TIR1 E3 ligase and degron tagging at the endogenous HSF-1 do not alter larval development while enabling efficient depletion of HSF-1 within 2 h of auxin treatment in adult animals (Edwards et al., 2021). Depletion of HSF-1 from Day 1 in young adults shortened the median lifespan by more than one third (Figure 1A and Table 1). Time-course analyses revealed that HSF-1 functions throughout the self-reproductive period in hermaphrodites to support lifespan (Table 1). On Day 5 of adulthood, when >98% of progenies were already produced (Supplementary Figure S1A), depletion of HSF-1 still resulted in a small yet significant lifespan shortening. It has been reported that auxin treatment itself extends lifespan in a concentration-dependent manner (Loose and Ghazi, 2021), and in one of our lifespan trials, we observed a modest lifespan extension when applying the standard 1 mM auxin to the control strain that expresses TIR1 but has no degron insertion at HSF-1 (Table 1, Trial #2). We, therefore, repeated the lifespan analyses using 0.5 mM auxin (Figure 1B). This experiment confirmed the results as using 1 mM auxin while it had much smaller effects on the control strain (Table 1, Trial #2). It is known that signals from the reproductive system could impact *C. elegans* lifespan (Hsin and Kenyon, 1999). Though depletion of HSF-1 from the adult germline dramatically reduced fecundity, depletion of HSF-1 from the soma had a modest impact on the brood size (Supplementary Figure S1B). Thus, our results suggest that HSF-1 functions directly through somatic maintenance to support lifespan. Collectively, our data determined the temporal requirement for HSF-1 in a normal lifespan, which overlaps with the self-reproductive period in early adulthood.

**TABLE 1:** The lifespan data and statistical test upon pan-somatic HSF-1 depletion in the wild-type background, related to Figure 1.

Trial	Strain, treatment	Median Lifespan (Days of adulthood)	S.E.	Observed/Total	% Lifespan change	*p*-value (Log rank)
#1, 20°C	CA1200 (eft-3p::tir1), control	15.69	0.38	89/101		
	CA1200 (eft-3p::tir1), 1mM auxin	14.92	0.35	83/99	−4.91	0.098
#2, 20°C	CA1200 (eft-3p::tir1), control	15.71	0.41	117/130		
	CA1200 (eft-3p::tir1), 1mM auxin	17.74	0.47	110/130	12.92	0.0004
	CA1200 (eft-3p::tir1), control	16.25	0.43	98/130		
	CA1200 (eft-3p::tir1), 0.5mM auxin	17.02	0.42	117/130	4.74	0.0453
#1, 20°C	JTL611 (eft-3p::tir1; hsf-1::degron), control	14.94	0.37	95/108		
	JTL611, 1mM auxin (Day 1)	9.87	0.11	90/120	−33.94	<1e-8
	JTL611, 1mM auxin (Day 2)	11.65	0.15	95/120	−22.02	<1e-8
	JTL611, 1mM auxin (Day 3)	12.84	0.17	102/120	−14.06	<1e-8
	JTL611, 1mM auxin (Day 4)	13.43	0.2	119/120	−10.11	4.4e-8
	JTL611, 1mM auxin (Day 5)	14.3	0.25	109/120	−4.28	0.0005
#2, 20°C	JTL611 (eft-3p::tir1; hsf-1::degron), control	16.39	0.39	94/100		
	JTL611, 0.5mM auxin (Day 1)	9.65	0.16	110/120	−41.12	<1e-8
	JTL611, 0.5mM auxin (Day 2)	12.77	0.16	116/120	−22.09	<1e-8
	JTL611, 0.5mM auxin (Day 3)	13.46	0.2	113/120	−17.88	<1e-8
	JTL611, 0.5mM auxin (Day 4)	14.56	0.22	117/120	−11.17	2.4e-7
	JTL611, 0.5mM auxin (Day 5)	15.05	0.29	112/120	−8.18	0.0006

### Long-Lived glp-1 and daf-2 Mutants Extend the Functional Period of HSF-1 in Lifespan Assurance

**FIGURE 2:**
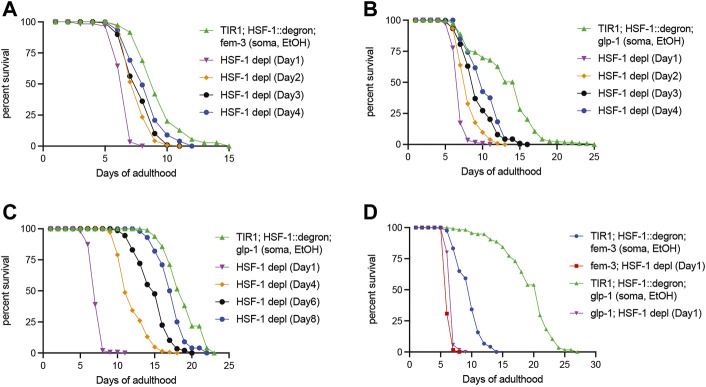
Temporal requirement for HSF-1 in lifespan extension by GSC arrest. **(A,B)** Lifespan analysis at 25°C upon pan-somatic depletion of HSF-1 by AID in the control *fem-3(q20)* **(A)** or the long-lived *glp-1(e2141)* background **(B)**. Animals were mock treated with ethanol (EtOH) from Day 1 of adulthood or treated with auxin starting at the indicated time. **(C,D)** Lifespan analysis at 25°C upon pan-somatic depletion of HSF-1 by AID when fed with carbenicillin-treated OP50 bacteria. Animals were mock treated with ethanol (EtOH) since Day 1 of adulthood as controls. HSF-1 depletion was initiated in the long-lived *glp-1(e2141)* background at the indicated time **(C)**, or a comparison was made between the *fem-3(q20)* and *glp-1(e2141)* when both had HSF-1 depleted since Day 1 of adulthood **(D)**.

HSF-1 is required for lifespan extension in multiple longevity pathways including those mediated by arrested germline stem cells (GSC) and reduced insulin/IGF-1 signaling (IIS). To understand the roles of HSF-1 in longevity pathways post-larval development, we performed lifespan analyses with HSF-1 depleted at different time points of adulthood in the long-lived *glp-1(e2141)* <arrested GSC> and *daf-2(e1370) <*reduced IIS > mutants. As a control for *glp-1(e2141),* we included another temperature-sensitive mutant *fem-3(q20)* that is sterile as *glp-1(e2141)* at the restricted temperature of 25°C *<* only producing sperm but not oocytes > but has a normal lifespan in the analyses. Similar to wild-type, HSF-1 is required in early adulthood (to at least Day 4 at 25°C) for the lifespan of *fem-3(q20),* and pan-somatic depletion of HSF-1 since Day 1 of adulthood shortened lifespan by about one third (Figure 2A; Table 2). HSF-1 makes a bigger contribution to the *glp-1(e2141)* lifespan, and its functional period is extended in *glp-1(e2141)* (Figure 2B; Table 2 Trial #1). These effects are more obvious when the median lifespan was further increased as animals were fed with antibiotic-treated bacteria (Figure 2C; Table 2 Trial #2 and #3). The additional lifespan extension was likely due to reduced bacterial infection since *glp-1(e2141)* delays later deaths with an atrophied pharynx but not earlier deaths from pharyngeal pathology by a bacterial infection (Zhao et al., 2017). In this growth condition, HSF-1 supported lifespan till at least Day 8, and depletion of HSF-1 in Day 1 adults resulted in >60% of lifespan reduction. It is noteworthy that depletion of HSF-1 was still effective in both the *fem-3 (q20)* and *glp-1(e2141)* animals on Day 4 (Supplementary Figures S2A, B), therefore, the relatively modest lifespan shortening effects in *fem-3(q20)* are not simply an artifact of defective AID during aging but rather suggest that HSF-1 is less active or its function is minimally required for the lifespan of *fem-3(q20)* beyond Day 4 at 25°C. While the signal of GSC arrest in *glp-1(e2141)* more than doubled the lifespan in the presence of HSF-1 (when fed with antibiotic-treated bacteria) (Figure 2D; Table 2, Trial #2), it had marginal effects if HSF-1 was depleted since Day 1 of young adults. These results indicate lifespan extension by arrested GSC is completely dependent on HSF-1 in adulthood, and provide an example that HSF-1’s roles in larval development and longevity are temporally uncoupled.

**TABLE 2:** The lifespan data and statistical test upon pan-somatic HSF-1 depletion in long-lived animals, related to Figure 2 and Supplementary Figure S2.

Trial without antibiotic	Strain, treatment	Median lifespan (Days of adulthood)	S.E.	Observed/Total	% Lifespan change	*p*-value (Log rank)
#1, 25°C	JTL623 (glp-1; eft-3p::tir1), control	13.42	0.56	115/120		
	JTL623 (glp-1; eft-3p::tir1), 1mM auxin (Day 1)	13.97	0.46	116/120	4.10	3.20E-01
	JTL667 (glp-1; eft-3p::tir1; hsf-1::degron), control	13.12	0.38	114/120		
	JTL667 (glp-1; eft-3p::tir1; hsf-1::degron), 1mM auxin (Day 1)	6.98	0.08	116/120	−46.80	<1e-8
	JTL667 (glp-1; eft-3p::tir1; hsf-1::degron), 1mM auxin (Day 2)	8.21	0.14	116/120	−37.42	<1e-8
	JTL667 (glp-1; eft-3p::tir1; hsf-1::degron), 1mM auxin (Day 3)	9.38	0.2	117/120	−28.51	<1e-8
	JTL667 (glp-1; eft-3p::tir1; hsf-1::degron), 1mM auxin (Day 4)	10.28	0.2	120/120	−21.65	<1e-8
#1, 25°C	JTL624 (fem-3; eft-3p::tir1), control	9.23	0.15	114/120		
	JTL624 (fem-3; eft-3p::tir1), 1mM auxin (Day 1)	9.15	0.17	115/120	−0.87	8.48E-01
	JTL670 (fem-3; eft-3p::tir1; hsf-1::degron), control	9.44	0.16	118/120		
	JTL670 (fem-3; eft-3p::tir1; hsf-1::degron), 1mM auxin (Day 1)	6.61	0.07	120/121	−29.98	<1e-8
	JTL670 (fem-3; eft-3p::tir1; hsf-1::degron), 1mM auxin (Day 2)	7.77	0.09	120/120	−17.69	<1e-8
	JTL670 (fem-3; eft-3p::tir1; hsf-1::degron), 1mM auxin (Day 3)	7.92	0.11	119/120	−16.10	<1e-8
	JTL670 (fem-3; eft-3p::tir1; hsf-1::degron), 1mM auxin (Day 4)	8.45	0.13	116/120	−10.49	1.30E-05

**Trial (with Carbenicillin)**	**Strain, treatment**	**Median lifespan (Days of adulthood)**	**S.E.**	**Observed/Total**	**% Lifespan change**	***p*-value (Log rank)**
#2, 25°C	JTL623 (glp-1; eft-3p::tir1), control	20.34	0.30	118/120		
	JTL623 (glp-1; eft-3p::tir1), 1mM auxin (Day 1)	20.72	0.53	61/61	1.87	8.92E-02
	JTL667 (glp-1; eft-3p::tir1; hsf-1::degron), control	19.33	0.36	115/119		
	JTL667 (glp-1; eft-3p::tir1; hsf-1::degron), 1mM auxin (Day 1)	6.87	0.05	120/120	−64.46	<1e-8
#3, 25°C	JTL667 (glp-1; eft-3p::tir1; hsf-1::degron), control	18.87	0.23	97/100		
	JTL667 (glp-1; eft-3p::tir1; hsf-1::degron), 1mM auxin (Day 1)	7.28	0.06	151/151	−61.42	<1e-8
	JTL667 (glp-1; eft-3p::tir1; hsf-1::degron), 1mM auxin (Day 4)	12.05	0.16	147/150	−36.14	<1e-8
	JTL667 (glp-1; eft-3p::tir1; hsf-1::degron), 1mM auxin (Day 6)	14.89	0.18	147/150	−21.09	<1e-8
	JTL667 (glp-1; eft-3p::tir1; hsf-1::degron), 1mM auxin (Day 8)	17.36	0.19	100/100	−8.00	4.90E-07
						
#2, 25°C	JTL624 (fem-3; eft-3p::tir1), control	9.16	0.17	121/121		
	JTL624 (fem-3; eft-3p::tir1), 1mM auxin (Day 1)	9.75	0.13	120/120	6.44	3.82E-02
	JTL670 (fem-3; eft-3p::tir1; hsf-1::degron), control	9.66	0.18	120/120		
	JTL670 (fem-3; eft-3p::tir1; hsf-1::degron), 1mM auxin (Day 1)	6.33	0.05	119/120	−34.47	<1e-8
**Trial (without antibiotic)**	**Strain, treatment**	**Median lifespan (Days of adulthood)**	**S.E.**	**Observed/Total**	**% Lifespan change**	***p*-value (Log rank)**
#4, 20°C	JTL618 (daf-2; eft-3p::tir1), control	32.92	1.57	84/99		
	JTL618 (daf-2; eft-3p::tir1), 1mM auxin (Day 1)	33.69	1.48	87/100	2.34	6.39E-01
	JTL641 (daf-2; eft-3p::tir1; hsf-1::degron), control	31.93	1.65	82/101		
	JTL641 (daf-2; eft-3p::tir1; hsf-1::degron), 1mM auxin (Day 5)	20.85	0.79	94/100	−34.70	<1e-8
	JTL641 (daf-2; eft-3p::tir1; hsf-1::degron), 1mM auxin (Day 7)	25.47	0.79	98/100	−20.23	3.60E-08
**Trial with Carbenicillin**	**Strain, treatment**	**Median lifespan (Days of adulthood)**	**S.E.**	**Observed/Total**	**% Lifespan change**	***p*-value (Log rank)**
#5, 20°C	JTL618 (daf-2; eft-3p::tir1), control	42.18	1.31	112/150		
	JTL618 (daf-2; eft-3p::tir1), 1mM auxin (Day 1)	43.41	1.25	100/150	2.92	8.10E-01
	JTL641 (daf-2; eft-3p::tir1; hsf-1::degron), control	40.88	1.14	98/150		
	JTL641 (daf-2; eft-3p::tir1; hsf-1::degron), 1mM auxin (Day 7)	27.07	0.67	93/150	−33.78	<1e-8
	JTL641 (daf-2; eft-3p::tir1; hsf-1::degron), 1mM auxin (Day 11)	29.85	0.48	142/151	−26.98	<1e-8
	JTL641 (daf-2; eft-3p::tir1; hsf-1::degron), 1mM auxin (Day 15)	32.59	0.55	121/126	−20.28	<1e-8
	JTL641 (daf-2; eft-3p::tir1; hsf-1::degron), 1mM auxin (Day 19)	39.22	0.52	116/119	−4.06	1.10E-07

We also tested the roles of HSF-1 in longevity by reducing IIS using the *daf-2(e1370)* mutant. Due to the high incidence of internal hatching upon HSF-1 depletion on Day 1 (54%, 189 out of 350) and Day 3 (39%, 136 out of 350) of *daf-2(e1370),* it is difficult to estimate the total contribution of HSF-1 to lifespan throughout adulthood. However, similar to the *glp-1(e2141)* mutant, *daf-2(e1370)* also seems to extend the functional period of HSF-1 as depletion of HSF-1 since Day 5 and Day 7 resulted in ∼35% and ∼20% of lifespan reduction in *daf-2(e1370)* (Supplementary Figure S2C, Table 2 Trial #4) while depletion of HSF-1 since Day 5 in the wild-type only led to modest (4–8%) lifespan reduction (Table 1). This becomes apparent when *daf-2(e1370)* animals were fed with antibiotic-treated bacteria, in which HSF-1 contributes to longevity up to Day 19 of adulthood (Supplementary Figure S2D, Table 2 Trial #5). Collectively, we found longevity by GSC arrest and reduced IIS correlates with extended functional period of HSF-1.

### HSF-1 Directly Activates Transcription From its Associated Promoters in Somatic Cells of Young Adults in the Absence of Heat Stress

**FIGURE 3:**
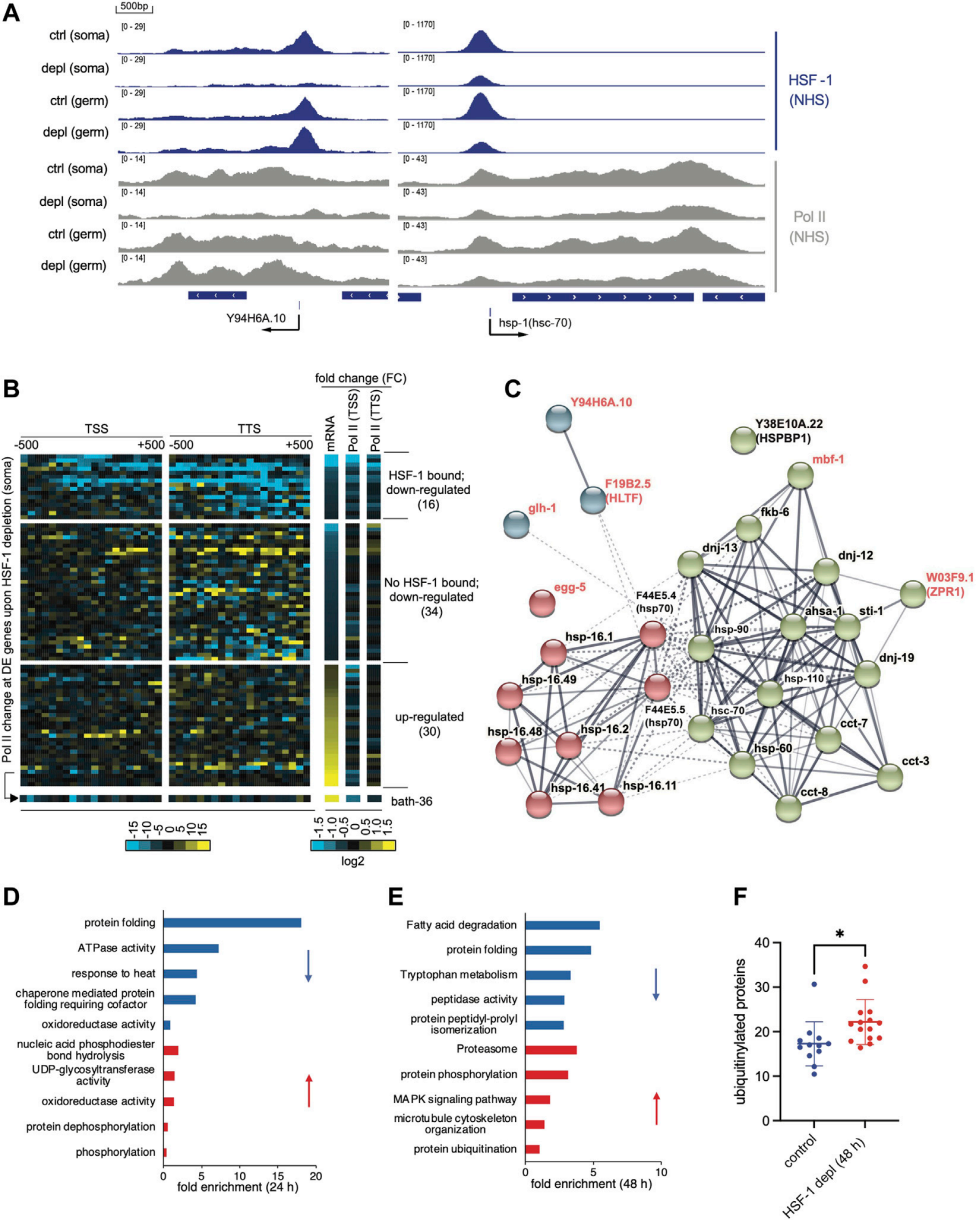
Transcriptional program of HSF-1 in the soma of young adults. **(A)** G browser views of HSF-1 and RNA Polymerase II (Pol II) occupancy at the *Y94H6A.10* and *hsp-1 (hsc-70)* gene loci in young adults grown at the ambient temperature of 20°C with either the mock treatment (ctrl) or an acute HSF-1 depletion (depl) from the soma (soma) or the germline (germ) (auxin treatment for 2 h). **(B)** Heatmaps of Pol II occupancy changes upon HSF-1 depletion in young adults from the soma for 2 h and mRNA changes by HSF-1 depletion for 24 h at differentially expressed (DE) genes. The data shown are based on ChIP-seq and RNA-seq experiments in JTL611 (wild-type background). The DE genes are those that significantly altered mRNA levels (FDR: 0.05) upon pan-somatic depletion of HSF-1 for 24 h (Supplementary Table S2). Pol II occupancy change was calculated as the difference of normalized ChIP-seq reads (HSF-1 depletion vs. the control) mapped in 50 bp bins, ±500 bp from the transcription start sites (TSS) and transcription termination site (TTS). The fold change (FC) of Pol II occupancy (2 h of HSF-1 depletion) and mRNA (24 h of HSF-1 depletion) are shown in the log2 scale. The DE genes were first ranked by fold change of mRNA and then by whether bound by HSF-1 at the promoters (1 kb from TSS). The number of DE genes in each group is shown in parentheses. **(C)** The gene network is directly activated by HSF-1 in the somatic cells from *glp-1(e2141)*. Genes included are those with HSF-1 binding peaks at the promoters and significantly decreased expression upon HSF-1 depletion from the soma for 8 h or 24 h on Day 1 of adulthood. The protein-protein interaction network was retrieved from the STRING database and grouped by kmeans clustering (*n* = 3). The node color represents the cluster to which the gene belongs. The color saturation of edges represents the confidence score of functional interaction. Genes with names in black encode chaperones or co-chaperones, and genes with names in red are those with other functions. **(D,E)** Gene Ontology (GO) analyses of DE genes by HSF-1 depletion from the soma in *glp-1(e2141)* for 24 h **(D)** and 48 h **(E)**. The top5 GO terms based on enrichment score are shown for down-regulated genes (blue bars) and up-regulated genes (red bars) respectively. **(F)** Quantification of immunofluorescence of ubiquitinylated proteins in the control group or upon HSF-1 depletion from the soma since Day 1 of adulthood for 48 h in *glp-1(e2141)*. Data are shown as mean ± standard deviation (*n*>=12). Only animals with clear staining by the control antibody against REC-8 were included in analyses. Statistical significance was calculated by unpaired, two-tailed Student’s t test. *p < 0.05.

The prevailing view is that HSF-1 promotes longevity through its ability to activate the HSR. HSF-1 also drives a transcriptional program that is different from the HSR in *C. elegans* larval development (Li et al., 2016). In addition, enhancing HSF-1 activities by transgenic over-expression of HSF-1, mild mitochondrial perturbation, or ablation of its negative regulator HSB-1 extends lifespan through functions beyond inducing the canonical HSR (Baird et al., 2014; Kumsta et al., 2017; Higuchi-Sanabria et al., 2018; Egge et al., 2019; Williams et al., 2020; Sural et al., 2020). To better understand the molecular mechanism underlying the physiological roles of HSF-1 in lifespan, we set out to determine the transcriptional program of endogenous HSF-1 in somatic cells on Day 1 of young adults. Recently, we have determined HSF-1 binding sites specifically in the soma or the germline and binding sites used in both tissue types through whole animal ChIP-seq analyses following an acute depletion of HSF-1 in the soma or germline (Edwards et al., 2021). Among those sites, 79 promoter-associated HSF-1 binding peaks are either enriched in the soma (e.g., *Y94H6A.10*) or shared by the soma and germline (e.g. *hsp-1/hsc-70*) (Figure 3A). This result suggests that albeit the number of binding peaks is smaller compared to that in larval development (Li et al., 2016), HSF-1 can bind to promoters without thermal stress in adult somatic cells that are postmitotic.

We then examined the transcriptional impact of HSF-1 binding *via* RNA-seq analyses following HSF-1 depletion from somatic cells by AID. We performed RNA-seq in the control strains that express TIR1 but have no degron insertion in a time course of auxin treatment (Supplementary Figures S3A–D). This set of experiments, when compared to those using the experimental strains with degron tagging at HSF-1, identified the small groups of differentially expressed (DE) genes caused by auxin treatment (Supplementary Figure S3A, mock *vs*. auxin treatment in the control strain) or by degron insertion at HSF-1 (Supplementary Figure S3B, compare the control and experimental strains with mock treatment). As auxin treatment resulted in almost identical transcriptional changes in the two control strains that express TIR1 in the soma and germline, respectively (Supplementary Figure S3C), we conclude that these changes were due to auxin rather than off-target effects of TIR1. By filtering out HSF-1-independent changes (Supplementary Figures S3A, B, Supplementary Table S1), our methods specifically determined DE genes caused by HSF-1 depletion (Supplementary Figure S3D). Among the 80 DE genes resulting from 24 h of HSF-1 depletion in the soma, 16 out of 50 down-regulated genes have HSF-1 binding at the promoter (Supplementary Figure S3E, Supplementary Table S2) while only 1 out of 34 up-regulated genes are bound by HSF-1, suggesting that HSF-1 functions as a transcriptional activator in somatic cells of young adults. The group of 16 HSF-1-bound down-regulated genes exhibit a decrease of RNA Polymerase II (Pol II) occupancy both at the promoter and at the end of genes (Figure 3B) upon 2 h depletion of HSF-1 suggesting that they are likely the direct targets of HSF-1 and that HSF-1 functions at the step of Pol II recruitment. For examples, a decrease of Pol II occurs across the genes of *Y94H6A.10* and *hsp-1/hsc-70* upon HSF-1 depletion in the soma (Figure 3A). On the contrary, down-regulated genes without HSF-1 binding and up-regulated genes (including the only HSF-1 bound gene, *bath-36* in this group) lack a correlation between mRNA (24 h post HSF-1 depletion) and Pol II occupancy changes (2 h of HSF-1 depletion), suggesting they are indirectly impacted by HSF-1 (Figure 3B).

### HSF-1 Drives Expression of an Important Sub-Chaperome in Fully-Developed Somatic Cells

Among the direct targets of HSF-1 in somatic tissues of young adults (Supplementary Figure S3F), most have substantial HSF-1 binding in the germline as well (*Y94H6A.10* is the only exception). To control for the potential interference by mRNA from the germline and increase the specificity and sensitivity of RNA-seq analyses, we also performed the experiments in the germline deficient *glp-1(e2141)* background. Indeed, we have uncovered a bigger group of HSF-1 direct targets (28 genes), each of which is associated with one of the 79 HSF-1 binding peaks at the promoter in the soma and decreases mRNA upon 8 h and/or 24 h of HSF-1 depletion (Figure 3C, Supplementary Table S2). Similar to the list of genes identified in the wild-type background, the majority of these genes (22 out of 28) (Figure 3C, gene names in black) encode either molecular chaperones or co-chaperones, which function together in protein folding and protein conformation maintenance. These chaperone/co-chaperone genes are grouped into two clusters with one including canonical HSR genes encoding members of HSP70 and small heat shock proteins (HSP-16) whose expression is highly inducible by stress (Figure 3C, cluster #1: red nodes), and the other cluster enriched with constitutively expressed chaperones and co-chaperones (Figure 3C, cluster #2: green nodes). The latter group contains the essential HSP-90 and HSC-70 chaperones as well as their co-chaperones (DNJs, FKB-6, STI-1, AHSA-1, HSP-110, and the HSPBP1 orthologue Y38E10A.22). It also contains subunits of the chaperonin complexes in the cytosol (CCT-3, CCT-7, and CCT-8) and mitochondria (HSP-60). These HSF-1-dependent, constitutively expressed chaperone and co-chaperone genes span most the ATP-dependent chaperone systems in metazoan (Clare and Saibil, 2013) and dictate protein folding capacity in the cytosol, nuclei, and mitochondria. It is noteworthy that the majority of genes in cluster #2 (14 out of 16) belong to the developmental HSF-1 transcriptional program (Li et al., 2016), which are regulated by HSF-1 differently from the HSR. As genetic perturbation of the HSP-90, HSC-70 and cytosolic chaperonin systems accelerates age-dependent proteostatic and physiological declines (Brehme et al., 2014), our data suggest a role of HSF-1 in longevity by activating the expression of these chaperone systems in fully-developed somatic cells.

A decrease of this selective ‘sub-chaperome’ precedes massive transcriptomic changes upon HSF-1 depletion in *glp-1(e2141),* which also supports that they are the primary targets of HSF-1 in adult somatic cells. 19 out of 22 HSF-1 directly regulated chaperone/co-chaperone genes already decrease expression at 8 h of HSF-1 depletion (Supplementary Table S2). They remain the most prominent functional group (‘protein folding’) among ∼400 DE genes at 24 h of HSF-1 depletion as shown by Gene Ontology (GO) analyses (note the enrichment of genes with ‘ATPase activity’ results from ATP-dependent chaperones, Figure 3D). 48 h of HSF-1 depletion, however, led to much bigger changes in the transcriptome (>1200 DE genes, Supplementary Table S2). Genes in the ubiquitin–proteasome system (UPS) are up-regulated (Figure 3E), which is a typical response to protein misfolding, implicating imbalanced proteome over time caused by loss of HSF-1 and consequent decline in protein folding. This idea is supported by the increased levels of ubiquitylated proteins in somatic tissues upon 48 h of HSF-1 depletion (Figure 3F, Supplementary Figures S3G, H), and is consistent with published results that HSF-1 RNAi leads to increased protein misfolding and aggregation (Nollen et al., 2004; Ben-Zvi et al., 2009).

We also identified a few nonchaperone genes as HSF-1 direct targets (Figure 3C, gene names in red), although their roles in lifespan are not clear. Two of the encoded proteins, MBF-1 (Multiprotein Bridging Factor 1) and W03F9.1/ZPR1 in cluster #2 are proposed to be transcriptional coactivators and interact with chaperones/co-chaperones based on the studies of their orthologues in other model systems (Arce et al., 2006; Kannan et al., 2020). Especially, the *mbf-1* gene is a direct target of HSF-1 in *C. elegans* larval development (Li et al., 2016), and expression of its orthologues in yeast and *Drosophila* is also controlled by HSF1 (Birch-Machin et al., 2005; Pincus et al., 2018). Similar to *mbf-1*, expression of *Y94H6A.10* (a gene with unknown function) and *F19B2.5/HLTF* (encodes an SWI/SNF chromatin remodeler family protein) is activated by HSF-1 in larval development, suggesting that at least part of HSF-1 developmental transcription program sustains in adulthood.

To test whether the transcriptional program of HSF-1 identified in *glp-1(e2141)* is unique to this longevity model and how much it is linked to the higher growth temperature (25 *vs*. 20°C for wild-type), we performed RNA-seq analyses in the *fem-3(q20)* control at 25°C. The *fem-3(q20)* shows a similar decrease of HSF-1 direct targets including constitutively expressed chaperones, stress-inducible chaperones as well as nonchaperone genes upon 24-h HSF-1 depletion (Supplementary Figures S3I, J). Although there is a trend of a bigger difference in *glp-1(e2141)* compared to that in *fem-3(q20)* (Supplementary Figure S3I), not all HSF-1 direct targets are more highly expressed in *glp-1(e2141)* as one may expect if HSF-1 is hyperactivated by GSC-mediated longevity signal (Supplementary Figure S3J). The mRNA of stress-inducible *hsp70* and *hsp-16.2* are at higher levels in *glp-1(e2141)* compared to *fem-3(q20)*, suggesting expression levels of these canonical HSR genes are not solely dictated by the growth temperature. On the contrary, higher mRNA levels of constitutively expressed chaperones are observed in *fem-3(q20).* This expression pattern is likely linked to the different cell compositions in these two types of animals. Though *fem-3(q20)* is sterile at 25°C, it still has a germline that makes sperm. The smaller changes upon depletion of HSF-1 from the soma in *fem-3(q20)* could be well explained by the ‘masking effect’ from unchanged mRNAs in the germline. Consistent with this idea, our recent paper shows that *hsc-70* and *hsp-90* mRNAs are enriched in the germline while HSR is more robust in the soma (Edwards et al., 2021), therefore *fem-3(q20)* with more germ cells shows higher levels of constitutive chaperones but lower levels of inducible chaperones in whole animal analyses. Collectively, the HSF-1 transcriptional program that we identified in the germline deficient *glp-1(e2141)* likely applies to somatic cells of animals with normal lifespan as seen in *fem-3(q20)*. Future studies are needed to determine whether HSF-1 activities are enhanced by longevity signals in the absence of external stress.

### Tissue Requirements for HSF-1 in Larval Development and Lifespan Assurance are Different

While most of the HSF-1-dependent, constitutively-expressed chaperones and co-chaperones have roles in all cell types, over-expression of HSF-1 in specific tissues (e.g., neural cells) is sufficient to extend lifespan (Morley and Morimoto, 2004; Douglas et al., 2015). To understand the tissue requirement for endogenous HSF-1 in lifespan, we have made transgenic models that express TIR1 E3 ligase specifically in one of the major somatic tissue types (neural system, intestine, muscle, and hypodermis) to enable tissue-specific depletion of HSF-1 by AID. All of our AID models successfully depleted HSF-1 from the nuclei in the target tissues within 2 h of auxin treatment (Supplementary Figures S4A–D). We also checked HSF-1:degron:GFP in the neighboring tissues and were able to confirm the specificity of AID except for our neural model, in which the nuclear HSF-1 seemed also depleted in intestinal cells near the head but not in the center or toward the tail (Supplementary Figure S4D). The same neural TIR1 transgene did not deplete degron:GFP (not fused with HSF-1) in intestinal cells near the head as it did for HSF-1:degron:GFP upon auxin treatment (Supplementary Figure S4E). This result implies depletion of HSF-1 from a subset of intestinal nuclei in our neural AID model is unlikely due to leaky expression of TIR1 but instead dependent on neural HSF-1. However, due to high levels of auto-florescence in the intestine, future studies with more sensitive and quantitative methods to measure HSF-1 protein levels and its localization, and using alternative neural AID models are needed to confirm the results.

**FIGURE 4:**
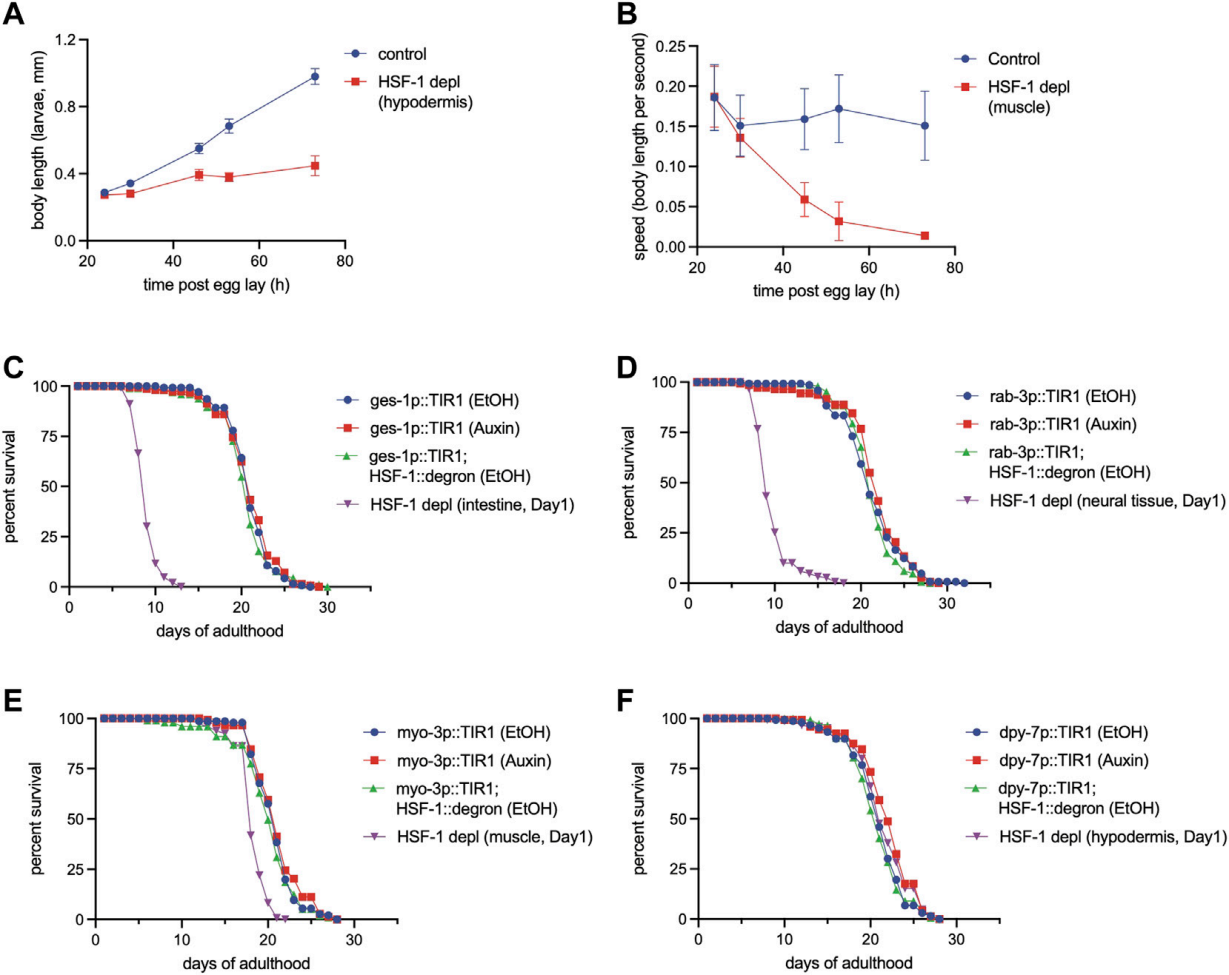
Different tissue requirements for HSF-1 in larval development and lifespan assurance. **(A)** Size tracking of developing larvae with continuous HSF-1 depletion in the hypodermis initiated at egg lay. Experiments were done in the wild-type background at 20°C. Data are represented as mean ± standard deviation (*n*>=12). *p* < 0.0001 (control vs. HSF-1 depletion, two-way ANOVA). **(B)** Mobility of developing larvae measured as body length per second with continuous HSF-1 depletion in the muscle initiated at egg lay. Experiments were done in the wild-type background at 20°C. Data are represented as mean ± standard deviation (*n*>=12). *p* < 0.0001 (control vs. HSF-1 depletion, two-way ANOVA). **(C–F)** Lifespan analysis at 25°C upon depletion of HSF-1 by AID in the intestine **(C)**, neural cells **(D)**, muscle **(E)**, and hypodermis **(F)** since Day 1 of adulthood. Experiments were done in the control strains (expressing TIR1 only) and HSF1 AID models (expressing TIR1 and having degron insertion at endogenous *hsf-1*) in the long-lived *glp-1(e2141)* background. Animals were fed with carbenicillin treated OP50 bacteria and were mock treated with ethanol (EtOH) or treated with auxin since Day 1 of adulthood.

We then tested the tissue requirements of HSF-1 for larval development. Loss of HSF-1 in any of these tissues since egg lay led to larval developmental arrest or delay (Figure 4A, Supplementary Figures S5A–C). HSF-1 depletion from hypodermis caused larval arrest at L3-L4, and the animals were associated with molting defects (Supplementary Figure S5D). Animals with HSF-1 depleted in the intestine, neural cells or muscle managed to develop into adults. Intestinal depletion of HSF-1 led to a huge larval delay, more than doubling the time needed for developing into adults (Supplementary Figure S5A). Loss of HSF-1 in neural cells and muscle had a relatively milder larval delay (Supplementary Figures S5B, C) but greatly reduced mobility eventually making the animals paralyzed at the young adult stage (Figure 4B, Supplementary Figure S5E). Depletion of HSF-1 in the muscle also led to egg-laying defects, resulting in 100% internal hatching (20/20 animals, Supplementary Figure S5F). It is noteworthy that none of the tissue-specific HSF-1 depletion phenocopied the L1-L2 arrest by pan-somatic depletion of HSF-1 (Edwards et al., 2021), suggesting that HSF-1 functions cooperatively in all the tissue types tested to support larval development.

We then performed lifespan analysis with tissue-specific depletion of HSF-1 on Day 1 of adulthood. We chose *glp-1(e2141)* as the model since pan-somatic depletion of HSF-1 in this background exhibited the biggest lifespan shortening effect, therefore, providing sufficient dynamic range to examine potentially smaller effects from HSF-1 depletion in a single tissue type. Depletion of HSF-1 in the intestine and neural cells both resulted in ∼55% lifespan shortening (Figure 4C, D, Table 3) which is only slightly smaller than that upon pan-somatic depletion (Figure 2C; Table 2). Depletion of HSF-1 from muscle led to a fairly small but still significant decrease (∼9%) in median lifespan (Figure 4E; Table 3). Despite the essentiality of HSF-1 in hypodermis during larval development, loss of HSF-1 from hypodermis post-larval development did not significantly alter lifespan (Figure 4F; Table 3). Collectively, our results indicate the different tissue requirements for HSF-1 in development and lifespan, suggesting that the roles of HSF-1 in these two processes are uncoupled.

**TABLE 3:** The lifespan data and statistical test upon tissue-specific HSF-1 depletion, related to Figure 4.

Targeted tissue (with Carbenicillin)	Strain, treatment	Median Lifespan (Days of adulthood)	S.E.	Observed/Total	% Lifespan change	*p*-value (Log rank)
Intestine, 25°C	JTL658 (glp-1; ges-1p::tir1), control	21	0.22	140/150		
	JTL658 (glp-1; ges-1p::tir1), 1mM auxin (Day 1)	20.96	0.27	147/155	−0.19	3.53E-01
	JTL700 (glp-1; ges-1p::tir1; hsf-1::degron), control	20.47	0.34	93/100		
	JTL700 (glp-1; ges-1p::tir1; hsf-1::degron), 1mM auxin (Day 1)	9.05	0.11	146/150	−55.79	<1e-8
Neural cells, 25°C	JTL659 (glp-1; rab-3p::tir1), control	21.24	0.3	145/150		
	JTL659 (glp-1; rab-3p::tir1), 1mM auxin (Day 1)	21.62	0.33	142/149	1.79	2.78E-01
	JTL701 (glp-1; rab-3p::tir1; hsf-1::degron), control	21.21	0.24	146/150		
	JTL701 (glp-1; rab-3p::tir1; hsf-1::degron), 1mM auxin (Day 1)	9.79	0.17	150/150	−53.84	<1e-8
Muscle, 25°C	JTL660 (glp-1; myo-3p::tir1), control	20.82	0.21	146/150		
	JTL660 (glp-1; myo-3p::tir1), 1mM auxin (Day 1)	21.14	0.23	143/150	1.54	2.20E-01
	JTL702 (glp-1; myo-3p::tir1; hsf-1::degron), control	19.99	0.36	97/100		
	JTL702 (glp-1; myo-3p::tir1; hsf-1::degron), 1mM auxin (Day 1)	18.22	0.16	145/150	−8.85	<1e-8
Hypodermis, 25°C	JTL661 (glp-1; dpy-7p::tir1), control	20.94	0.27	140/150		
	JTL661 (glp-1; dpy-7p::tir1), 1mM auxin (Day 1)	21.95	0.28	142/150	4.82	3.10E-03
	JTL703 (glp-1; dpy-7p::tir1; hsf-1::degron), control	20.84	0.25	144/150		
	JTL703 (glp-1; dpy-7p::tir1; hsf-1::degron), 1mM auxin (Day 1)	21.46	0.29	145/150	2.98	2.72E-02

## Discussion

HSF-1 is known as a prominent lifespan promoting factor in *C. elegans* and has been proposed to contribute to lifespan largely from its activities at the larval stages based on RNAi experiments (Volovik et al., 2012) and through its ability to activate the HSR to cope with proteotoxic stress and maintain proteostasis (Hsu et al., 2003; Morley and Morimoto, 2004; Hansen et al., 2005; Cohen et al., 2006; Shemesh et al., 2013; Labbadia and Morimoto, 2015). HSF-1 is also required for larval development through a transcriptional program different from the HSR (Li et al., 2016). Therefore, it is important to distinguish the role of HSF-1 in the maintenance of adult somatic cells, which are all postmitotic, with that in development, since defects in both processes could shorten lifespan.

In this study, we used the auxin-inducible degron (AID) system to enable rapid and efficient depletion of HSF-1 and determined the spatiotemporal requirement for HSF-1 in lifespan post-larval development. We found that HSF-1 is predominantly required during the early adulthood to support lifespan, which overlaps with the self-reproductive period in wild-type animals (Figure 1; Table 1). This temporal correlation is interesting, implicating that somatic maintenance by HSF-1 may be coupled with reproductive activities to ensure a favorable environment for internal embryonic development and successful egg-laying. Supporting this idea, we have observed egg retention and increased internal hatching upon HSF-1 depletion from pan-soma or specifically from the muscle. It is not clear though whether HSF-1 has lost most of its activities after the reproductive period or the molecular decline has reached a threshold then so that the protective mechanism by HSF-1 makes no consequence. The functional period of HSF-1 in lifespan is extended in long-lived animals with GSC arrest or reduced IIS (Figure 2; Table 2). Importantly, GSC-arrest mediated lifespan extension is completely dependent on HSF-1 activity in adulthood (Figure 2D). This result suggests the functional impacts of HSF-1 in larval development and longevity can be uncoupled temporally, and also implies that germline signals in adults may regulate HSF-1’s activities in the soma. Our RNA-seq analysis on long-lived *glp-1(2141)* and the control *fem-3(q20)* to compare HSF-1 activities is inconclusive due to the different cell compositions of these animals. Future work with tissue-specific transcription measurement is needed to test whether longevity signaling (e.g., GSC and IIS) hyperactivates HSF-1 in physiological condition (not upon stress) and/or sustains HSF-1 activities longer during aging. Consistent with the idea that functions of HSF-1 in development and longevity can be uncoupled, we found the tissue requirements for HSF-1 in larval development and lifespan are different (Figure 4). Loss of HSF-1 in the intestine or neural system showed >80% of lifespan shortening effects as the pan-somatic depletion of HSF-1. As neural cells and intestine are the endocrine centers in *C. elegans*, it is likely that functions of HSF-1 in neural cells and intestine impact neighboring tissues nonautonomously as well. One complication of our results is that despite using the classic *rab-3* promoter to express TIR1 in the neural system, auxin treatment depleted HSF-1 from a subset of intestinal nuclei as well. Given the very different phenotypes in larval development, we do not believe that neural depletion of HSF-1 in our AID models affected physiology solely through intestinal HSF-1. However, it calls for future studies to further confirm the specificity of our neural model, and understand the interaction of HSF-1 in the intestine and nervous system.

In this study, we also examined the transcriptional program of HSF-1 and its regulation in somatic tissues of young adults by RNA-seq and ChIP-seq analyses following HSF-1 depletion. With careful control experiments, we have determined the differentially expressed (DE) genes induced by auxin. This gene list (Supplementary Table S1) not only helped us identify transcriptomic changes specific to HSF-1 depletion but also provides a useful reference for any transcriptomic studies using AID. We have found that HSF-1 activates transcription at its associated promoters, and functions at the step of Pol II recruitment (Figures 3A, B). This is different from the HSR in *Drosophila* and mammalian cells, where HSF-1 functions at releasing promoter-proximally paused Pol II into productive elongation (Duarte et al., 2016; Mahat et al., 2016). It is also different from the HSR in *C. elegans,* in which HSF-1 promotes both Pol II recruitment and elongation as depletion of HSF-1 either decreases Pol II across the gene (e.g., inducible *hsp70s*) or causes Pol II accumulation at the promoters (e.g. *hsp-110*) during heat shock (Edwards et al., 2021). The different roles of HSF-1 in transcription regulation in physiology and the HSR may be due to the fact that Pol II pausing or stalling is not prevalent in physiological conditions in *C. elegans* as it lacks the pausing factor NELF (Kruesi et al., 2013; Maxwell et al., 2014).

Diverse mechanisms have been proposed for HSF-1’s roles in longevity (Baird et al., 2014; Kumsta et al., 2017; Higuchi-Sanabria et al., 2018; Egge et al., 2019; Williams et al., 2020; Sural et al., 2020), and the HSF-1 direct target genes that we identified in adult soma provide a molecular basis for understanding functions of endogenous HSF-1 in comparison to gain-of-function phenotypes or pleotropic effects. One example is that expression of the troponin protein PAT-10 is activated and responsible for longevity by HSF-1 overexpression (16). We did not find that HSF-1 binds to the *pat-10* promoter or depletion of HSF-1 alters its expression, implicating regulation of PAT-10 is likely unique to HSF-1 overexpression. HSF-1 directly activates a compact transcriptional program including classical stress-inducible chaperones, constitutively-expressed chaperones, and co-chaperones as well as a few nonchaperone genes (Figure 3C). We conclude that protein folding is the primary function of this HSF-1 transcriptional program because 1 > chaperone and co-chaperone genes take more than three quarters of this group (22 out of 28), 2 > their expressions change early upon HSF-1 depletion, and 3 > transcriptomic signature at a later time (48 h after HSF-1 depletion) suggests proteotoxic stress response as a consequence of losing folding capacity (Figures 3D, E). Despite the higher fold changes in expression of certain inducible chaperones upon HSF-1 depletion (Supplementary Figure S3G), the mRNA levels of constitutively-expressed chaperones are much higher (Supplementary Figure S3H). For example, the mRNA of *hsc-70* is ∼50 fold as the inducible hsp70, *F44E5.5* in the presence of HSF-1. Thus, despite some differences in biochemical property, it is reasonable to think the constitutively expressed HSC-70 may have a bigger contribution to the overall folding capacity than the inducible HSP70 in the absence of stress. It is important to note that this group of constitutively-expressed chaperones and co-chaperones (cluster #2 in Figure 3C) overlap largely with the evolutionarily conserved ‘core chaperome’ defined by a previous study (in all the 5 functional groups and 50% of individual proteins) (Brehme et al., 2014). Their expression decline was proposed to underlie human brain aging, and genetic perturbation of this ‘core chaperome’ led to the early onset of proteome imbalance and healthspan shortening in *C. elegans* (Brehme et al., 2014). Collectively, we propose that HSF-1’s role in lifespan assurance is primarily through activating the expression of a selective group of chaperone and co-chaperone genes and enhancing protein folding capacity.

HSF-1 also directly activates a few nonchaperone genes. Of those, MBF-1, W03F9.1/ZPR1, and F19B2.5/HLTF are proposed to have roles in transcription regulation and might be involved in the secondary transcriptional response to HSF-1 depletion. Future studies will examine whether they also affect animal lifespan, and understand the biological significance of their regulation by HSF-1.

## Materials and Methods

### Worm Strains and Maintenance

Unless stated, *C. elegans* strains were maintained at 20°C on NGM plates seeded with OP50 bacteria and were handled using standard techniques (Brenner, 1974). The temperature-sensitive *glp-1 (e2141) and fem-3(q20)* animals were maintained at 15°C and grown at 25°C (since L1) for experiments.

The HSF-1 AID models were made by CRISPR knock-in of aid:gfp to the C-terminus of endogenous *hsf-1* gene as detailed in our previous publication (Edwards et al., 2021). New tissue-specific TIR1 models were made by modification of CA1200 (*eft-3p:tir1:mRuby*) and swapping the *eft-3* promoter with tissue-specific promoters. We first removed the *eft-3* promoter through microinjection of two chemically modified synthetic sgRNA (Synthego) in CA1200, which are against the upstream and downstream regions of the *eft-3* promoter, along with Cas9 Nuclease (Integrated DNA Technologies, IDT) following the previously published protocol (Prior et al., 2017). The DNA sequences targeted by the sgRNAs are GCT​CTG​GTA​CCC​TCT​AGT​CA (upstream) and AGT​TAC​GGT​CCT​TGT​CGA​GT (downstream) respectively. The resulting promoter-less allele contains a short insertion (‘GGCATCCA’) between the two cutting sites. We then inserted the tissue-specific promoters by microinjection of an sgRNA against that short insertion in the promoter-less allele (corresponding DNA sequence: GGT​CCT​TGT​TGG​ATG​CCT​CA) with Cas9 Nuclease and PCR fragments of tissue-specific promoters as the repair templates. The *rab-3b* (1.2 kb), *dpy-7* (350 bp), and *myo-3* (2.5 kb) promoters were used for the expression of TIR1 in neural cells, hypodermis, and muscle. The published allele in CA1209 (*ges-1p:tir1:mRuby*) was used for intestinal expression of TIR1. All the transgenic models made by CRISPR were outcrossed 6 times before use.

### Auxin Treatment

Auxin treatment was performed by transferring worms to bacteria-seeded NGM plates containing 1 mM (if not specified) or 0.5 mM auxin (Auxin: indole-3-acetic acid, Sigma). The preparation of auxin stock solution (400 mM in ethanol) and auxin-containing NGM plates was performed as previously described (Zhang et al., 2015). In all experiments, worms were also transferred to NGM plates containing 0.25% or 0.125% of ethanol (EtOH) to serve as the mock-treated control for 1 and 0.5 mM auxin respectively.

### Measurements of Body Length, Mobility, and Brood Size

The HSF-1 AID animals and the corresponding control animals that only express TIR1 were age-synchronized by egg lay for 1 h on EtOH or Auxin plates. Larvae were grown for the indicated time (Figure 4 and Supplementary Figure S4) and crawling animals were recorded using a Leica M205 FA microscope. Videos were imported into ImageJ and analyzed for the size of animals (body length, mm) and mobility (body length per second, BLPS) using the wrMTrck plugin.

For brood size analyses (Supplementary Figure S1), animals were synchronized by egg lay and singled at L4/young adult stage onto plates containing ethanol (control) or auxin (HSF-1 depl) to lay eggs for 24 h. Worms were then transferred to new plates every day and eggs were allowed to hatch and grow to the L3 stage, at which point the number of progeny was counted.

### Lifespan Assays and Antibiotic Treatment of OP50

Age-synchronized worms were scored as dead and removed in the absence of touch response or pharyngeal pumping 6 days per week. For fertile animals, worms were transferred to fresh plates every day through the reproductive period to remove progeny. Bagged, desiccated, or missing animals were censored from analysis.

In a subset of lifespan experiments, carbenicillin treatment of OP50 was performed as previously described to prevent bacterial growth (Lenaerts et al., 2008). Briefly, freshly grown *E. coli* OP50 cells were spun at 3000 × g for 20 min and resuspended in the same volume of M9 buffer supplemented with 0.5 mM of carbenicillin. The bacteria were then incubated in the shaker at 37°C for 3 h. The bacteria were concentrated by 5 fold (pelleting the cells, removing 80% M9 buffer, and resuspending) and seeded onto 10 cm NGM plates using 400 ul of concentrated bacteria per plate.

### Brightfield and Fluorescence Imaging

Imaging of live animals was done by immobilizing age-synchronized young adult animals in a drop of M9 buffer containing 6 mM levamisole on a 2% agarose pad. Fluorescent images (Supplementary Figure S2) were acquired immediately using a Zeiss LSM880 Confocal Microscope with a ×40 water objective. Zen software was used to obtain z-stacks and subsequent processing. Brightfield images (Supplementary Figure S4) were taken on a Zeiss axio observer inverted microscope with a ×20 air objective.

### RNA Extraction and RNA-Seq

The HSF-1 AID animals and the corresponding control animals that only express TIR1 were synchronized by treatment of alkaline hypochlorite solution (bleach). The experiments in JTL611, JTL621 (HSF-1 AID models), and CA1200, CA1199 (control) were done as described in our recent paper (Edwards et al., 2021). For the experiments using the set of HSF-1 AID strains in *glp-1 (e2141)* and *fem-3(q20)* backgrounds, synchronized L1 larvae were grown for 48 h at 25°C on 10 cm normal NGM plates (∼500 worms per plate) to develop into young adults. Approximately 120 young adult worms were picked onto 10 cm NGM plates containing either EtOH or auxin and kept for an indicated time before collection (8, 24, and 48 h). For each condition, RNA was extracted using a 300 μL Trizol reagent. Worms were vortexed continuously for 20 min at 4°C and then went through one cycle of freeze-thaw to help release RNA. Following this, RNA was purified using the Direct-zol RNA MiniPrep kit (Zymo Research) as per the manufacturer’s instructions using on column DNase I digestion to remove genomic DNA.

Total RNAs were polyA enriched, and directional RNA-seq libraries were prepared using the NEBNext Ultra II RNA library prep Kit. Paired-end sequencing was done at a NovaSeq 6,000 sequencer at OMRF clinical genomics core. The majority of samples were sequenced by 50 bp. A subset of samples that were sequenced with longer reads was trimmed to 50 bp to make all downstream mapping and analyses consistent.

### RNA-Seq and ChIP-Seq Data Analyses

RNA-seq analyses were conducted as previously described (Edwards et al., 2021). Briefly, RNA-seq reads were mapped to the Ensembl WBcel235 genome using RNA STAR (Dobin et al., 2013). The mapped reads were then subject to FeatureCounts in Rsubread (Liao et al., 2019) for quantification. Differential expression (DE) analyses were then done using edgeR (Robinson et al., 2010) with default settings except for using Likelihood Ratio Test and filtering out those lowly expressed genes with CPM (counts per million) value less than 1 in more than 75% samples. Gene ontology analysis (GO) of DE genes was conducted using the program DAVID (http://david.abcc.ncifcrf.gov/) with functional annotation clustering to collapse redundant GO terms. The enrichment score for each cluster was shown.

ChIP-seq reads mapping, peak calling, and generation of genomic occupancy files (bedgraph files) were detailed in our recent publication (Edwards et al., 2021). To assign HSF-1 ChIP-seq peaks to promoters, transcription start sites (TSSs) determined by GRO-cap (Kruesi et al., 2013) were used where available. To visualize and compare the ChIP-seq data in genome browser views, the bedgraph files were normalized to reads per million using MACS2 call peak -B –SPMR and visualized using Integrative Genomics Viewer (IGV) (Robinson et al., 2011) with WS235 genome. Quantification of genomic occupancy was done by mapping the center of ChIP fragments to a reference point (e.g. HSF-1 peak summits) using windowBed in bedtools (Quinlan and Hall, 2010) and Matrix in R. For quantitative comparison of Pol II occupancy between conditions, all Pol II ChIP-seq data was normalized to 8 million reads, corresponding to the lowest coverage after duplicate filtering among all conditions. Heatmaps were generated with the Java TreeView package (Saldanha, 2004).

### Immunofluorescence

Worms were prepared by freeze-cracking and fixed in 4% paraformaldehyde, as described (Charlie et al., 2006) with the following changes. JTL667 (*glp-1; eft-3p:tir1; hsf-1:degron*) were synchronized by bleach synchronization, and L1 larvae were grown on NGM plates containing ethanol at 25°C for 48 h. Young adult worms were rinsed off in M9 buffer and plated on either ethanol or auxin plates for 48 h before rinsing for freeze-cracking. A methanol incubation for 15 min at 4°C after fixation was added to eliminate the TIR1:RFP signal in the worms. The samples were co-stained with Anti-Ubiquitinylated proteins (clone FK2, mouse, Sigma Cat# 04-263) at 1:200 and anti-REC-8 (rabbit, Novus Cat# 49230002) at 1:100 at 15°C overnight. We then labeled FK2 with anti-mouse-Alexa 647 (Invitrogen Cat# A32728) and anti-REC-8 with goat anti-rabbit-Alexa 488 (Invitrogen Cat# A32731) by incubating for 2 h at room temperature.

## Statistical Analysis

Statistical significance was calculated by unpaired, two-tailed Student’s t test in Microsoft excel or two-way ANOVA comparison in GraphPad Prism. Lifespan statistics were calculated by Log-rank test using OASIS2 online lifespan analysis software (https://sbi.postech.ac.kr/oasis2/). RNA-seq and ChIP-seq analyses are described in Materials And Methods.

## Data Availability

The datasets presented in this study can be found in online repositories. The names of the repository/repositories and accession number(s) can be found below: GEO - GSE162067.

## References

[B1] Arce D. P. Tonon C. Zanetti M. E. Godoy A. V. Hirose S. Casalongue C. A. (2006). The Potato Transcriptional Co-Activator StMBF1 Is Up-Regulated in Response to Oxidative Stress and Interacts with the TATA-Box Binding Protein. BMB Rep. 39 (4), 355–360. 10.5483/bmbrep.2006.39.4.355 16889677

[B2] Baird N. A. Douglas P. M. Simic M. S. Grant A. R. Moresco J. J. Wolff S. C. (2014). HSF-1-Mediated Cytoskeletal Integrity Determines Thermotolerance and Life Span. Science 346 (6207), 360–363. 10.1126/science.1253168 25324391 PMC4403873

[B3] Balch W. E. Morimoto R. I. Dillin A. Kelly J. W. (2008). Adapting Proteostasis for Disease Intervention. Science 319 (5865), 916–919. 10.1126/science.1141448 18276881

[B4] Ben-Zvi A. Miller E. A. Morimoto R. I. (2009). Collapse of Proteostasis Represents an Early Molecular Event in *Caenorhabditis elegans* Aging. Proc. Natl. Acad. Sci. U.S.A. 106 (35), 14914–14919. 10.1073/pnas.0902882106 19706382 PMC2736453

[B5] Birch-Machin I. Gao S. Huen D. McGirr R. White R. A. Russell S. (2005). Genomic Analysis of Heat-Shock Factor Targets in Drosophila. Genome Biol. 6 (7), R63. 10.1186/gb-2005-6-7-r63 15998452 PMC1175994

[B6] Brehme M. Voisine C. Rolland T. Wachi S. Soper J. H. Zhu Y. (2014). A Chaperome Subnetwork Safeguards Proteostasis in Aging and Neurodegenerative Disease. Cell Rep. 9 (3), 1135–1150. 10.1016/j.celrep.2014.09.042 25437566 PMC4255334

[B7] Brenner S. (1974). The Genetics of *Caenorhabditis elegans* . Genetics 77 (1), 71–94. 10.1093/genetics/77.1.71 4366476 PMC1213120

[B8] Charlie N. K. Schade M. A. Thomure A. M. Miller K. G. (2006). Presynaptic UNC-31 (CAPS) Is Required to Activate the Gαs Pathway of the *Caenorhabditis elegans* Synaptic Signaling Network. Genetics 172 (2), 943–961. 10.1534/genetics.105.049577 16272411 PMC1456257

[B9] Clare D. K. Saibil H. R. (2013). ATP-Driven Molecular Chaperone Machines. Biopolymers 99 (11), 846–859. 10.1002/bip.22361 23877967 PMC3814418

[B10] Cohen E. Bieschke J. Perciavalle R. M. Kelly J. W. Dillin A. (2006). Opposing Activities Protect Against Age-Onset Proteotoxicity. Science 313 (5793), 1604–1610. 10.1126/science.1124646 16902091

[B11] Dobin A. Davis C. A. Schlesinger F. Drenkow J. Zaleski C. Jha S. (2013). STAR: Ultrafast Universal RNA-Seq Aligner. Bioinformatics 29 (1), 15–21. 10.1093/bioinformatics/bts635 23104886 PMC3530905

[B12] Douglas P. M. Baird N. A. Simic M. S. Uhlein S. McCormick M. A. Wolff S. C. (2015). Heterotypic Signals from Neural HSF-1 Separate Thermotolerance from Longevity. Cell Rep. 12 (7), 1196–1204. 10.1016/j.celrep.2015.07.026 26257177 PMC4889220

[B13] Duarte F. M. Fuda N. J. Mahat D. B. Core L. J. Guertin M. J. Lis J. T. (2016). Transcription Factors GAF and HSF Act at Distinct Regulatory Steps to Modulate Stress-Induced Gene Activation. Genes Dev. 30 (15), 1731–1746. 10.1101/gad.284430.116 27492368 PMC5002978

[B14] Edwards S. L. Erdenebat P. Morphis A. C. Kumar L. Wang L. Chamera T. (2021). Insulin/IGF-1 Signaling and Heat Stress Differentially Regulate HSF1 Activities in Germline Development. Cell Rep. 36 (9), 109623. 10.1016/j.celrep.2021.109623 34469721 PMC8442575

[B15] Egge N. Arneaud S. L. B. Wales P. Mihelakis M. McClendon J. Fonseca R. S. (2019). Age-Onset Phosphorylation of a Minor Actin Variant Promotes Intestinal Barrier Dysfunction. Dev. Cell 51 (5), 587–601. 10.1016/j.devcel.2019.11.001 31794717 PMC6897307

[B16] Gomez-Pastor R. Burchfiel E. T. Neef D. W. Jaeger A. M. Cabiscol E. McKinstry S. U. (2017). Abnormal Degradation of the Neuronal Stress-Protective Transcription Factor HSF1 in Huntington's Disease. Nat. Commun. 8, 14405. 10.1038/ncomms14405 28194040 PMC5316841

[B17] Gomez-Pastor R. Burchfiel E. T. Thiele D. J. (2018). Regulation of Heat Shock Transcription Factors and Their Roles in Physiology and Disease. Nat. Rev. Mol. Cell Biol. 19 (1), 4–19. 10.1038/nrm.2017.73 28852220 PMC5794010

[B18] Hansen M. Hsu A. L. Dillin A. Kenyon C. (2005). New Genes Tied to Endocrine, Metabolic, and Dietary Regulation of Lifespan from a *Caenorhabditis elegans* Genomic RNAi Screen. PLoS Genet. 1 (1), 119–128. 10.1371/journal.pgen.0010017 16103914 PMC1183531

[B19] Higuchi-Sanabria R. Paul J. W. 3rd Durieux J. Benitez C. Frankino P. A. Tronnes S. U. (2018). Spatial Regulation of the Actin Cytoskeleton by HSF-1 During Aging. MBoC 29 (21), 2522–2527. 10.1091/mbc.e18-06-0362 30133343 PMC6254583

[B20] Hsin H. Kenyon C. (1999). Signals from the Reproductive System Regulate the Lifespan of *C. elegans* . Nature 399 (6734), 362–366. 10.1038/20694 10360574

[B21] Hsu A.-L. Murphy C. T. Kenyon C. (2003). Regulation of Aging and Age-Related Disease by DAF-16 and Heat-Shock Factor. Science 300 (5622), 1142–1145. 10.1126/science.1083701 12750521

[B22] Kannan A. Jiang X. He L. Ahmad S. Gangwani L. (2020). ZPR1 Prevents R-Loop Accumulation, Upregulates SMN2 Expression and Rescues Spinal Muscular Atrophy. Brain 143 (1), 69–93. 10.1093/brain/awz373 31828288 PMC6935747

[B23] Kim E. Wang B. Sastry N. Masliah E. Nelson P. T. Cai H. (2016). NEDD4-Mediated HSF1 Degradation Underlies α-Synucleinopathy. Hum. Mol. Genet. 25 (2), 211–222. 10.1093/hmg/ddv445 26503960 PMC4706110

[B24] Kruesi W. S. Core L. J. Waters C. T. Lis J. T. Meyer B. J. (2013). Condensin Controls Recruitment of RNA Polymerase II to Achieve Nematode X-Chromosome Dosage Compensation. Elife 2, e00808. 10.7554/eLife.00808 23795297 PMC3687364

[B25] Kumsta C. Chang J. T. Schmalz J. Hansen M. (2017). Hormetic Heat Stress and HSF-1 Induce Autophagy to Improve Survival and Proteostasis in *C. elegans* . Nat. Commun. 8, 14337. 10.1038/ncomms14337 28198373 PMC5316864

[B26] Labbadia J. Morimoto R. I. (2015). Repression of the Heat Shock Response Is a Programmed Event at the Onset of Reproduction. Mol. Cell 59 (4), 639–650. 10.1016/j.molcel.2015.06.027 26212459 PMC4546525

[B27] Lenaerts I. Walker G. A. Van Hoorebeke L. Gems D. Vanfleteren J. R. (2008). Dietary Restriction of *Caenorhabditis elegans* by Axenic Culture Reflects Nutritional Requirement for Constituents Provided by Metabolically Active Microbes. Journals Gerontology Ser. A Biol. Sci. Med. Sci. 63 (3), 242–252. 10.1093/gerona/63.3.242 PMC4333221 18375873

[B28] Li J. Chauve L. Phelps G. Brielmann R. M. Morimoto R. I. (2016). E2F Coregulates an Essential HSF Developmental Program that Is Distinct from the Heat-Shock Response. Genes Dev. 30 (18), 2062–2075. 10.1101/gad.283317.116 27688402 PMC5066613

[B29] Li J. Labbadia J. Morimoto R. I. (2017). Rethinking HSF1 in Stress, Development, and Organismal Health. Trends Cell Biol. 27 (12), 895–905. 10.1016/j.tcb.2017.08.002 28890254 PMC5696061

[B30] Liao Y. Smyth G. K. Shi W. (2019). The R Package Rsubread Is Easier, Faster, Cheaper and Better for Alignment and Quantification of RNA Sequencing Reads. Nucleic Acids Res. 47 (8), e47. 10.1093/nar/gkz114 30783653 PMC6486549

[B31] Loose J. A. Ghazi A. (2021). Auxin Treatment Increases Lifespan in *Caenorhabditis elegans* . Biol. Open 10 (5). 10.1242/bio.058703 PMC8186727 34184729

[B32] López-Otín C. Blasco M. A. Partridge L. Serrano M. Kroemer G. (2013). The Hallmarks of Aging. Cell 153 (6), 1194–1217. 10.1016/j.cell.2013.05.039 23746838 PMC3836174

[B33] Mahat D. B. Salamanca H. H. Duarte F. M. Danko C. G. Lis J. T. (2016). Mammalian Heat Shock Response and Mechanisms Underlying its Genome-Wide Transcriptional Regulation. Mol. Cell 62 (1), 63–78. 10.1016/j.molcel.2016.02.025 27052732 PMC4826300

[B34] Maxwell C. S. Kruesi W. S. Core L. J. Kurhanewicz N. Waters C. T. Lewarch C. L. (2014). Pol II Docking and Pausing at Growth and Stress Genes in *C. elegans* . Cell Rep. 6 (3), 455–466. 10.1016/j.celrep.2014.01.008 24485661 PMC4026043

[B35] Merkling S. H. Overheul G. J. van Mierlo J. T. Arends D. Gilissen C. van Rij R. P. (2015). The Heat Shock Response Restricts Virus Infection in Drosophila. Sci. Rep. 5, 12758. 10.1038/srep12758 26234525 PMC4522674

[B36] Morley J. F. Morimoto R. I. (2004). Regulation of Longevity in *Caenorhabditis elegans *by Heat Shock Factor and Molecular Chaperones. MBoC 15 (2), 657–664. 10.1091/mbc.e03-07-0532 14668486 PMC329286

[B37] Morton E. A. Lamitina T. (2013). *Caenorhabditis* *elegans* HSF-1 Is an Essential Nuclear Protein that Forms Stress Granule-Like Structures Following Heat Shock. Aging Cell 12 (1), 112–120. 10.1111/acel.12024 23107491 PMC3552056

[B38] Nollen E. A. A. Garcia S. M. van Haaften G. Kim S. Chavez A. Morimoto R. I. (2004). Genome-Wide RNA Interference Screen Identifies Previously Undescribed Regulators of Polyglutamine Aggregation. Proc. Natl. Acad. Sci. U.S.A. 101 (17), 6403–6408. 10.1073/pnas.0307697101 15084750 PMC404057

[B39] Pierce A. Podlutskaya N. Halloran J. J. Hussong S. A. Lin P.-Y. Burbank R. (2013). Over-Expression of Heat Shock Factor 1 Phenocopies the Effect of Chronic Inhibition of TOR by Rapamycin and Is Sufficient to Ameliorate Alzheimer's-Like Deficits in Mice Modeling the Disease. J. Neurochem. 124 (6), 880–893. 10.1111/jnc.12080 23121022 PMC6762020

[B40] Pierce A. Wei R. Halade D. Yoo S.-E. Ran Q. Richardson A. (2010). A Novel Mouse Model of Enhanced Proteostasis: Full-Length Human Heat Shock Factor 1 Transgenic Mice. Biochem. Biophysical Res. Commun. 402 (1), 59–65. 10.1016/j.bbrc.2010.09.111 20920476

[B41] Pincus D. Anandhakumar J. Thiru P. Guertin M. J. Erkine A. M. Gross D. S. (2018). Genetic and Epigenetic Determinants Establish a Continuum of Hsf1 Occupancy and Activity across the Yeast Genome. MBoC 29 (26), 3168–3182. 10.1091/mbc.e18-06-0353 30332327 PMC6340206

[B42] Prior H. Jawad A. K. MacConnachie L. Beg A. A. (2017). Highly Efficient, Rapid and Co-CRISPR-independent Genome Editing in *Caenorhabditis elegans* . G3 (Bethesda) 7 (11), 3693–3698. 10.1534/g3.117.300216 28893845 PMC5677160

[B43] Quinlan A. R. Hall I. M. (2010). BEDTools: A Flexible Suite of Utilities for Comparing Genomic Features. Bioinformatics 26 (6), 841–842. 10.1093/bioinformatics/btq033 20110278 PMC2832824

[B44] Robinson J. T. Thorvaldsdóttir H. Winckler W. Guttman M. Lander E. S. Getz G. (2011). Integrative Genomics Viewer. Nat. Biotechnol. 29 (1), 24–26. 10.1038/nbt.1754 21221095 PMC3346182

[B45] Robinson M. D. McCarthy D. J. Smyth G. K. (2010). edgeR: A Bioconductor Package for Differential Expression Analysis of Digital Gene Expression Data. Bioinformatics 26 (1), 139–140. 10.1093/bioinformatics/btp616 19910308 PMC2796818

[B46] Saldanha A. J. (2004). Java Treeview-Eextensible Visualization of Microarray Data. Bioinformatics 20 (17), 3246–3248. 10.1093/bioinformatics/bth349 15180930

[B47] Shemesh N. Shai N. Ben-Zvi A. (2013). Germline Stem Cell Arrest Inhibits the Collapse of Somatic Proteostasis Early in *Caenorhabditis elegans* adulthood. Aging Cell 12 (5), 814–822. 10.1111/acel.12110 23734734

[B48] Sural S. Liang C. Y. Wang F. Y. Ching T. T. Hsu A. L. (2020). HSB-1/HSF-1 Pathway Modulates Histone H4 in Mitochondria to Control mtDNA Transcription and Longevity. Sci. Adv. 6 (43). 10.1126/sciadv.aaz4452 PMC7577724 33087356

[B49] Vihervaara A. Sistonen L. (2014). HSF1 at a Glance. J. Cell Sci. 127 (Pt 2), 261–266. 10.1242/jcs.132605 24421309

[B50] Volovik Y. Maman M. Dubnikov T. Bejerano-Sagie M. Joyce D. Kapernick E. A. (2012). Temporal Requirements of Heat Shock Factor-1 for Longevity Assurance. Aging Cell 11 (3), 491–499. 10.1111/j.1474-9726.2012.00811.x 22360389 PMC4349560

[B51] Williams R. Laskovs M. Williams R. I. Mahadevan A. Labbadia J. (2020). A Mitochondrial Stress-Specific Form of HSF1 Protects Against Age-Related Proteostasis Collapse. Dev. Cell 54 (6), 758–772. 10.1016/j.devcel.2020.06.038 32735771

[B52] Zhang L. Ward J. D. Cheng Z. Dernburg A. F. (2015). The Auxin-Inducible Degradation (AID) System Enables Versatile Conditional Protein Depletion in *C. elegans* . Development 142 (24), 4374–4384. 10.1242/dev.129635 26552885 PMC4689222

[B53] Zhao Y. Gilliat A. F. Ziehm M. Turmaine M. Wang H. Ezcurra M. (2017). Two Forms of Death in Ageing *Caenorhabditis elegans* . Nat. Commun. 8, 15458. 10.1038/ncomms15458 28534519 PMC5457527

