# Erratum: Auxin-inducible degron system reveals temporal-spatial roles of HSF-1 and its transcriptional program in lifespan assurance

**DOI:** 10.3389/fragi.2022.1006781

**Published:** 2022-09-12

**Authors:** Frontiers Production Office

**Affiliations:** Frontiers Media SA, Lausanne, Switzerland

**Keywords:** aging, proteostasis, heat shock factor, auxin-inducible degron, temporal-spatial function, core chaperome, lifespan

Due to a production error, there are two errors in the first column of Table 2: In the column heading of trial 5, “Trial (without antibiotic)” instead of “Trial (with Carbenicillin)”, and in the second trial “#3,, 25°C” instead of “#3, 25°C”.

**TABLE 2:** A correction has been made to the section Results.

Trial (without antibiotic)	Strain, treatment	Median lifespan (days of adulthood)	S.E.	Observed/total	% Lifespan Change	*p*-value (log rank)
#1, 25°C	JTL623 (glp-1; eft-3p::tir1), control	13.42	0.56	115/120		
	JTL623 (glp-1; eft-3p::tir1), 1 mM auxin (Day 1)	13.97	0.46	116/120	4.10	3.20E-01
	JTL667 (glp-1; eft-3p::tir1; hsf-1::degron), control	13.12	0.38	114/120		
	JTL667 (glp-1; eft-3p::tir1; hsf-1::degron), 1 mM auxin (Day 1)	6.98	0.08	116/120	−46.80	<1e-8
	JTL667 (glp-1; eft-3p::tir1; hsf-1::degron), 1 mM auxin (Day 2)	8.21	0.14	116/120	−37.42	<1e-8
	JTL667 (glp-1; eft-3p::tir1; hsf-1::degron), 1 mM auxin (Day 3)	9.38	0.2	117/120	−28.51	<1e-8
	JTL667 (glp-1; eft-3p::tir1; hsf-1::degron), 1 mM auxin (Day 4)	10.28	0.2	120/120	−21.65	<1e-8
#1, 25°C	JTL624 (fem-3; eft-3p::tir1), control	9.23	0.15	114/120		
	JTL624 (fem-3; eft-3p::tir1), 1 mM auxin (Day 1)	9.15	0.17	115/120	−0.87	8.48E-01
	JTL670 (fem-3; eft-3p::tir1; hsf-1::degron), control	9.44	0.16	118/120		
	JTL670 (fem-3; eft-3p::tir1; hsf-1::degron), 1 mM auxin (Day 1)	6.61	0.07	120/121	−29.98	<1e-8
	JTL670 (fem-3; eft-3p::tir1; hsf-1::degron), 1 mM auxin (Day 2)	7.77	0.09	120/120	−17.69	<1e-8
	JTL670 (fem-3; eft-3p::tir1; hsf-1::degron), 1 mM auxin (Day 3)	7.92	0.11	119/120	−16.10	<1e-8
	JTL670 (fem-3; eft-3p::tir1; hsf-1::degron), 1 mM auxin (Day 4)	8.45	0.13	116/120	−10.49	1.30E-05

Trial (with Carbenicillin)	Strain, treatment	Median Lifespan (days of adulthood)	S.E.	Observed/total	% lifespan change	*p*-value (log rank)
#2, 25 °C	JTL623 (glp-1; eft-3p::tir1), control	20.34	0.30	118/120		
	JTL623 (glp-1; eft-3p::tir1), 1 mM auxin (Day 1)	20.72	0.53	61/61	1.87	8.92E-02
	JTL667 (glp-1; eft-3p::tir1; hsf-1::degron), control	19.33	0.36	115/119		
	JTL667 (glp-1; eft-3p::tir1; hsf-1::degron), 1 mM auxin (Day 1)	6.87	0.05	120/120	−64.46	<1e-8
#3, 25 °C	JTL667 (glp-1; eft-3p::tir1; hsf-1::degron), control	18.87	0.23	97/100		
	JTL667 (glp-1; eft-3p::tir1; hsf-1::degron), 1 mM auxin (Day 1)	7.28	0.06	151/151	−61.42	<1e-8
	JTL667 (glp-1; eft-3p::tir1; hsf-1::degron), 1 mM auxin (Day 4)	12.05	0.16	147/150	−36.14	<1e-8
	JTL667 (glp-1; eft-3p::tir1; hsf-1::degron), 1 mM auxin (Day 6)	14.89	0.18	147/150	−21.09	<1e-8
	JTL667 (glp-1; eft-3p::tir1; hsf-1::degron), 1 mM auxin (Day 8)	17.36	0.19	100/100	−8.00	4.90E-07
#2, 25 °C	JTL624 (fem-3; eft-3p::tir1), control	9.16	0.17	121/121		
	JTL624 (fem-3; eft-3p::tir1), 1 mM auxin (Day 1)	9.75	0.13	120/120	6.44	3.82E-02
	JTL670 (fem-3; eft-3p::tir1; hsf-1::degron), control	9.66	0.18	120/120		
	JTL670 (fem-3; eft-3p::tir1; hsf-1::degron), 1 mM auxin (Day 1)	6.33	0.05	119/120	−34.47	<1e-8

Trial (without antibiotic)	Strain, treatment	Median lifespan (days of adulthood)	S.E.	Observed/total	% lifespan change	*p*-value (log rank)
#4, 20 °C	JTL618 (daf-2; eft-3p::tir1), control	32.92	1.57	84/99		
	JTL618 (daf-2; eft-3p::tir1), 1 mM auxin (Day 1)	33.69	1.48	87/100	2.34	6.39E-01
	JTL641 (daf-2; eft-3p::tir1; hsf-1::degron), control	31.93	1.65	82/101		
	JTL641 (daf-2; eft-3p::tir1; hsf-1::degron), 1 mM auxin (Day 5)	20.85	0.79	94/100	−34.70	<1e-8
	JTL641 (daf-2; eft-3p::tir1; hsf-1::degron), 1 mM auxin (Day 7)	25.47	0.79	98/100	−20.23	3.60E-08

Trial (with Carbenicillin)	Strain, treatment	Median lifespan (days of adulthood)	S.E.	Observed/total	% lifespan change	*p*-value (log rank)
#5, 20 °C	JTL618 (daf-2; eft-3p::tir1), control	42.18	1.31	112/150		
	JTL618 (daf-2; eft-3p::tir1), 1 mM auxin (Day 1)	43.41	1.25	100/150	2.92	8.10E-01
	JTL641 (daf-2; eft-3p::tir1; hsf-1::degron), control	40.88	1.14	98/150		
	JTL641 (daf-2; eft-3p::tir1; hsf-1::degron), 1 mM auxin (Day 7)	27.07	0.67	93/150	−33.78	<1e-8
	JTL641 (daf-2; eft-3p::tir1; hsf-1::degron), 1 mM auxin (Day 11)	29.85	0.48	142/151	−26.98	<1e-8
	JTL641 (daf-2; eft-3p::tir1; hsf-1::degron), 1 mM auxin (Day 15)	32.59	0.55	121/126	−20.28	<1e-8
	JTL641 (daf-2; eft-3p::tir1; hsf-1::degron), 1 mM auxin (Day 19)	39.22	0.52	116/119	−4.06	1.10E-07

The publisher apologizes for this mistake. The original version of this article has been updated. 

