# Supplementary figures and images for: Auxin-Inducible Degron System Reveals Temporal-Spatial Roles of HSF-1 and Its Transcriptional Program in Lifespan Assurance
Source: Front Aging. 2022 Jul 11;3:899744. doi: 10.3389/fragi.2022.899744 (PMC9309338; doi:10.3389/fragi.2022.899744)

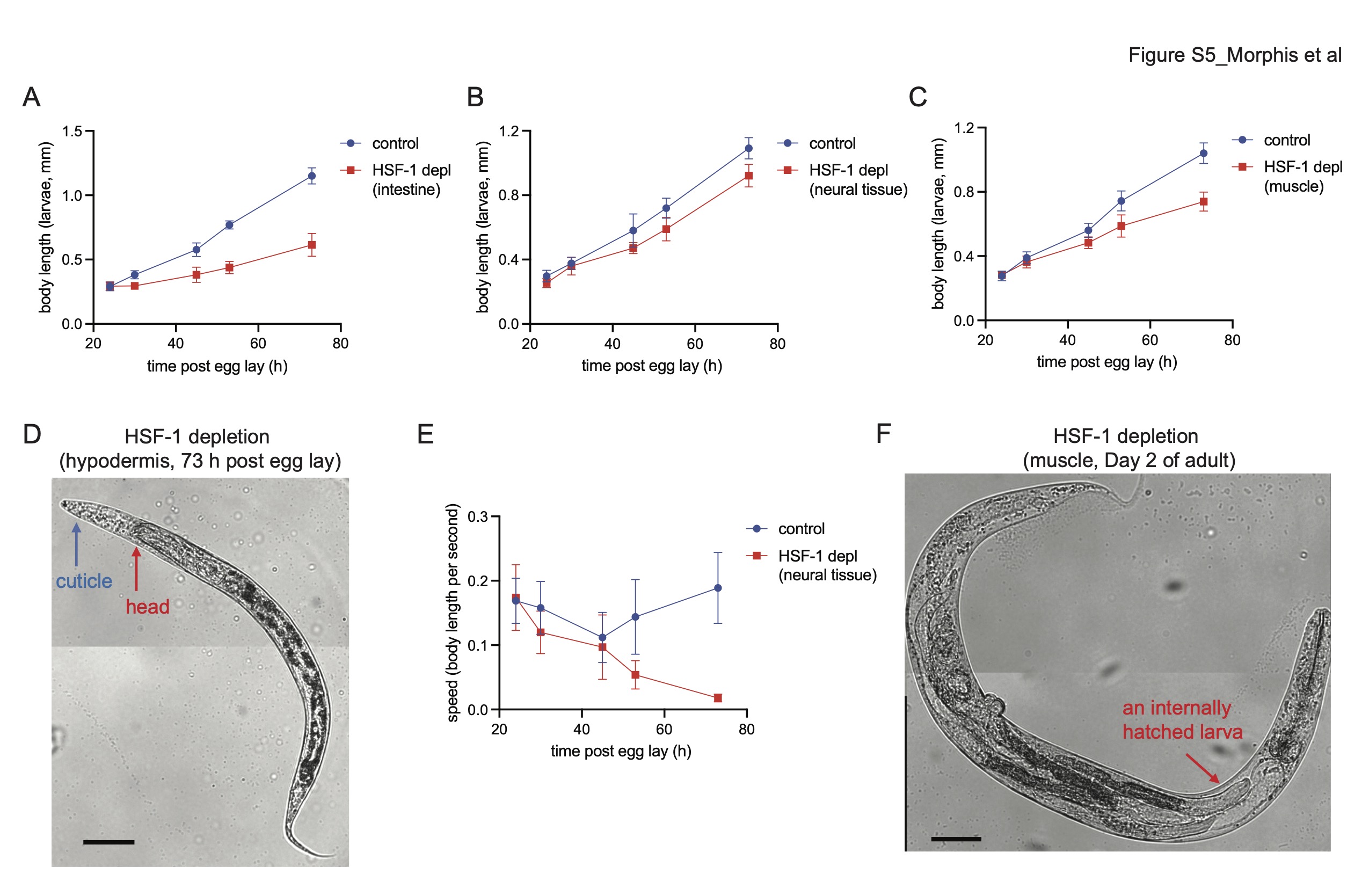

Supplement: Supplementary file 2 [file Image5.jpg]

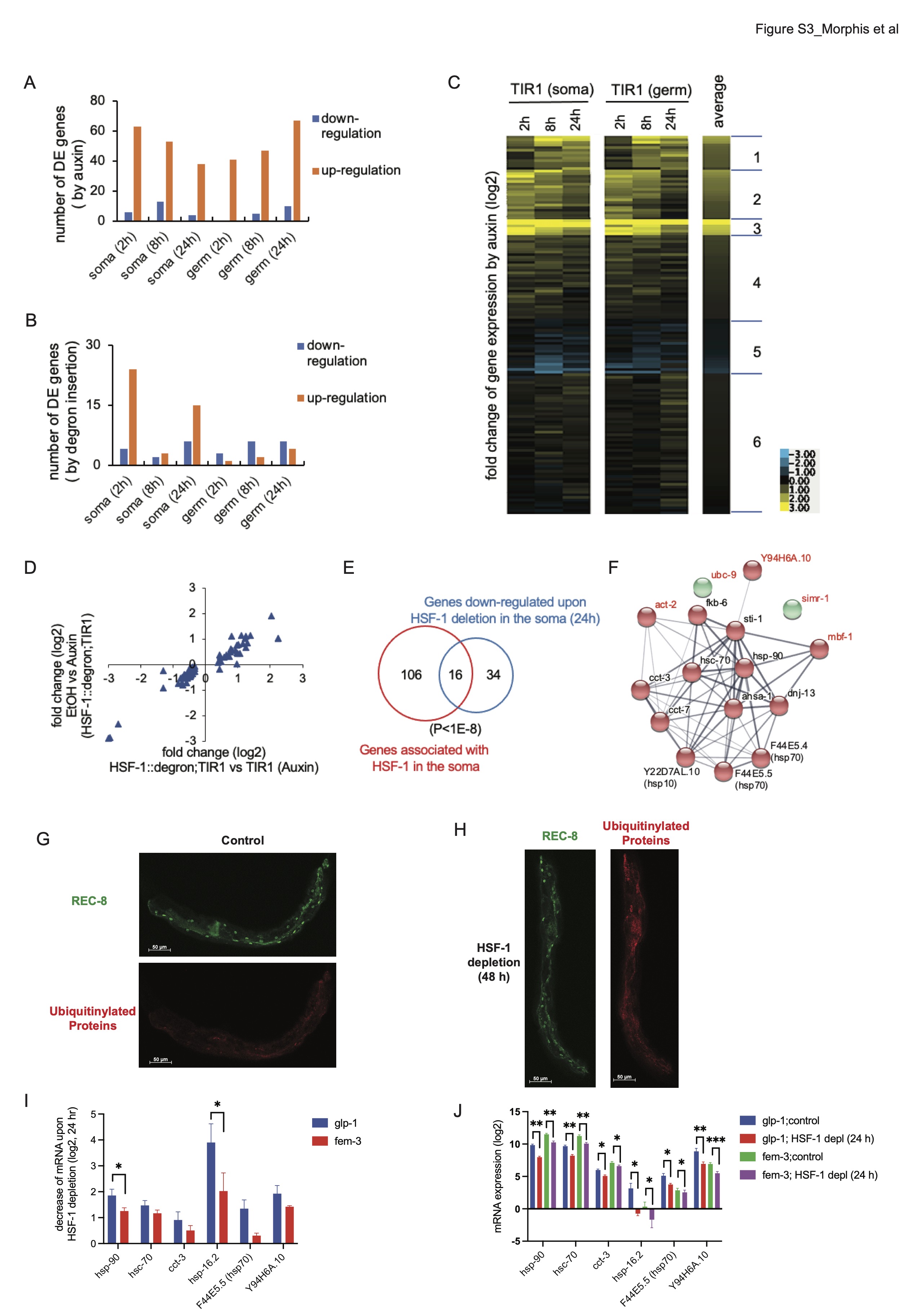

Supplement: Supplementary file 3 [file Image3.jpg]

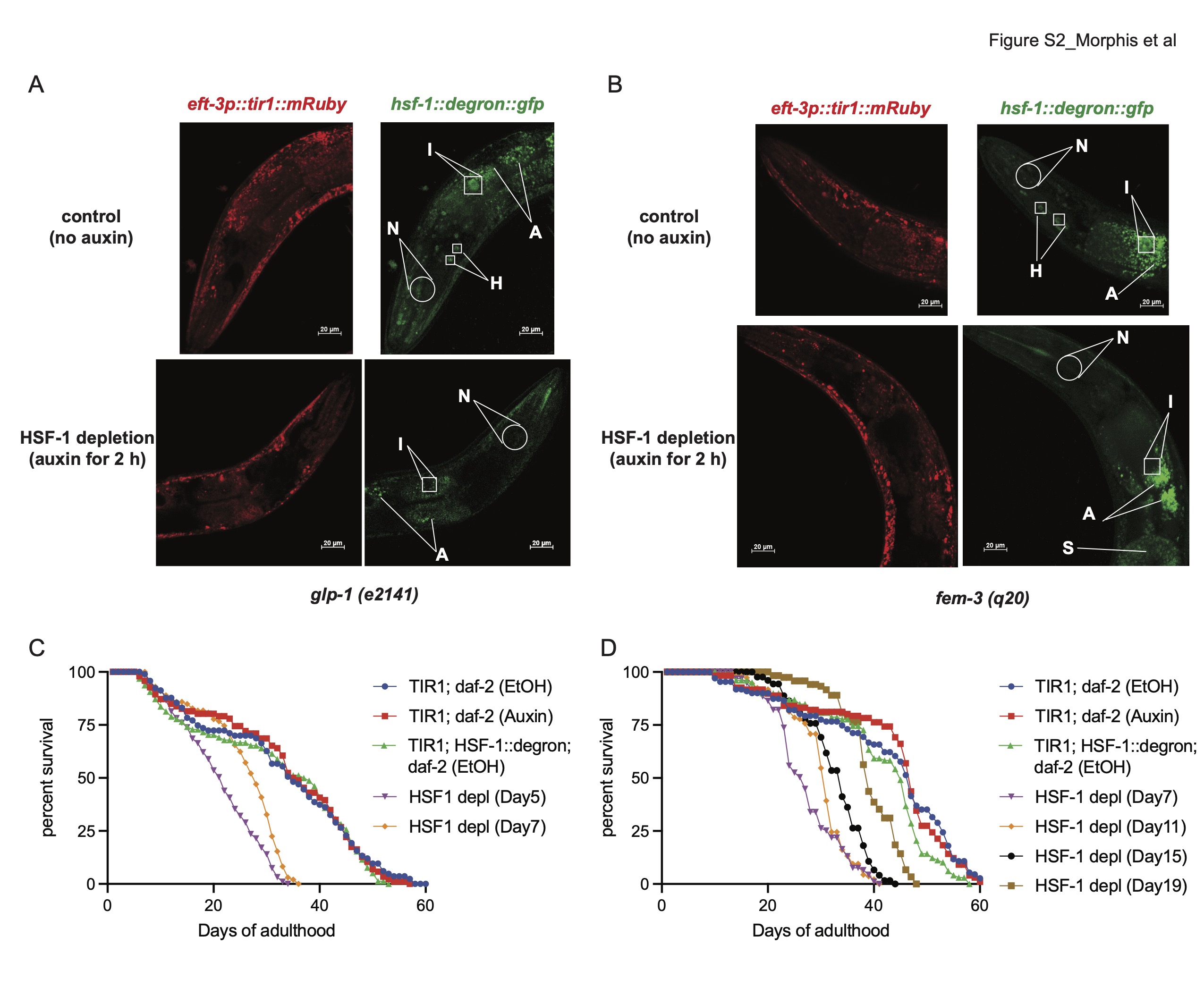

Supplement: Supplementary file 4 [file Image2.jpg]

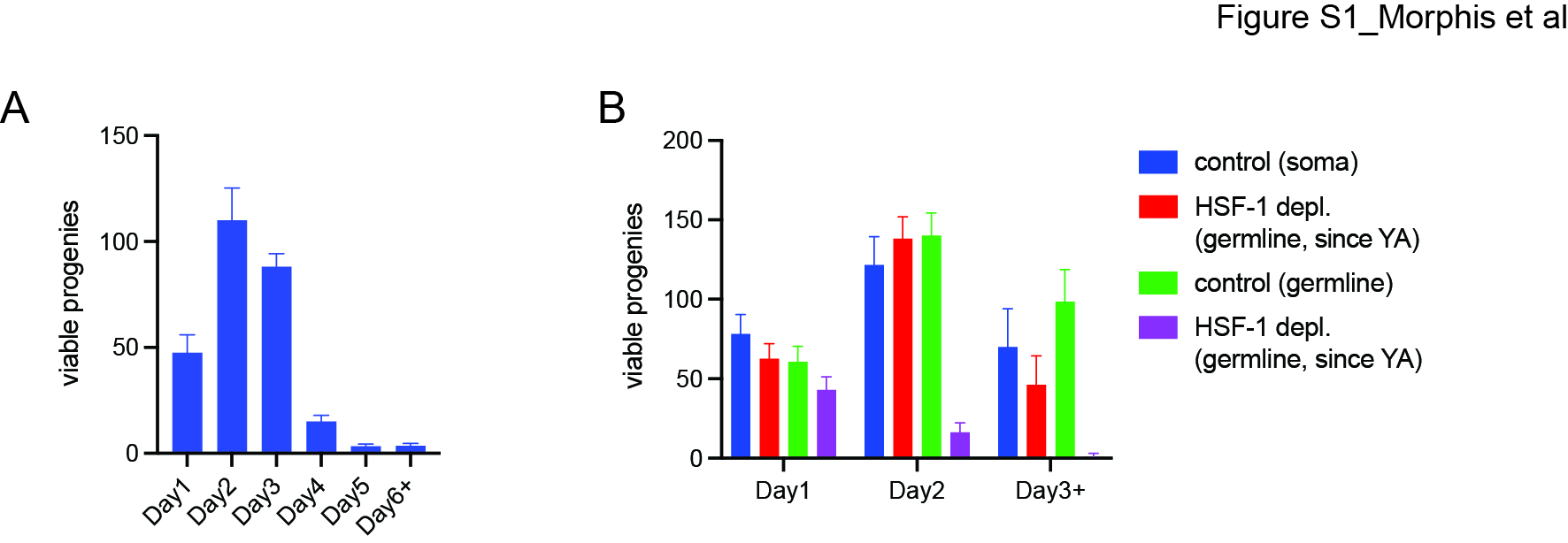

Supplement: Supplementary file 5 [file Image1.TIF]

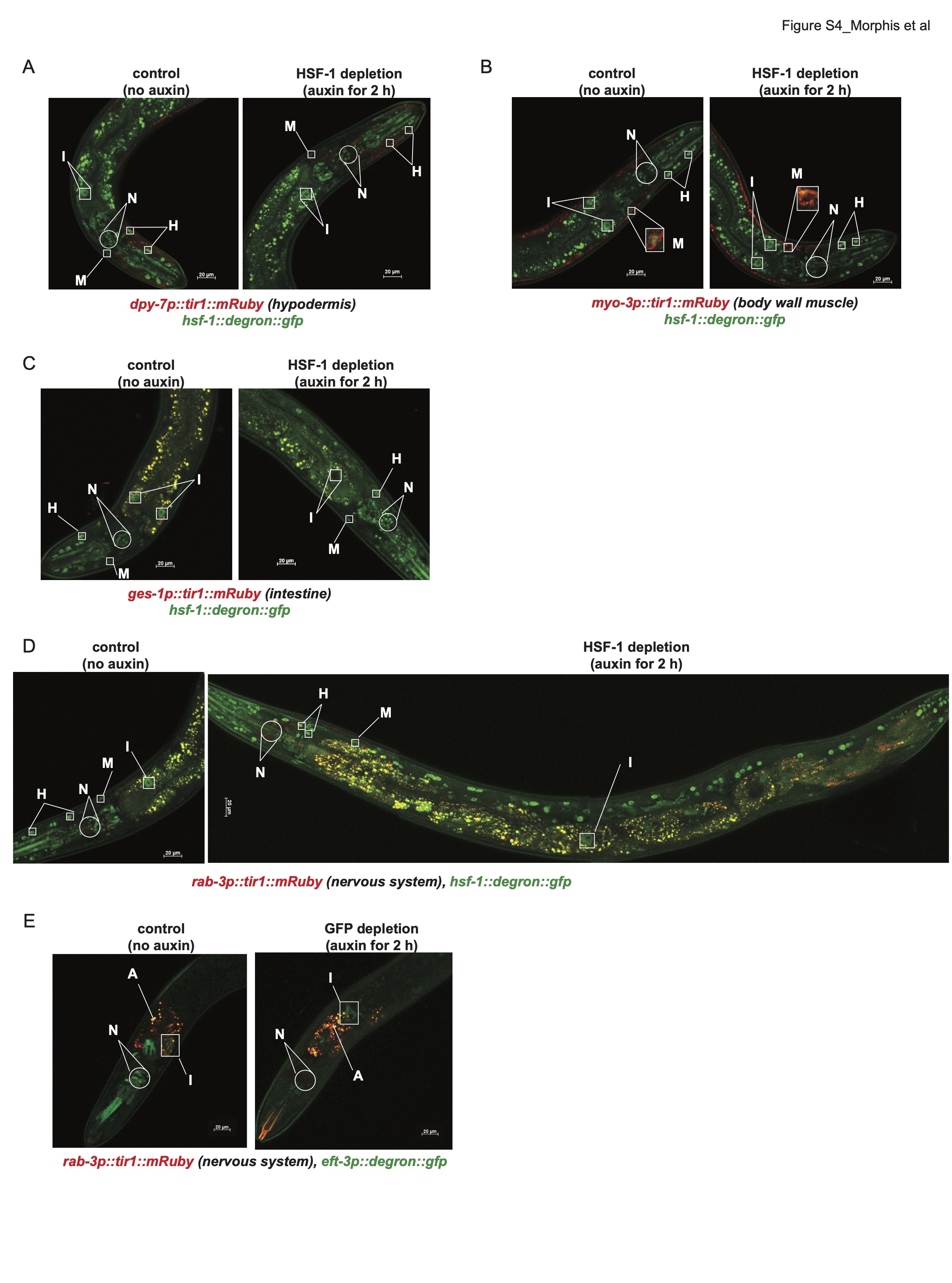

Supplement: Supplementary file 8 [file Image4.jpg]
