# Supplementary material for: Auxin-Inducible Degron System Reveals Temporal-Spatial Roles of HSF-1 and Its Transcriptional Program in Lifespan Assurance
Source: Front Aging. 2022 Jul 11;3:899744. doi: 10.3389/fragi.2022.899744 (PMC9309338; doi:10.3389/fragi.2022.899744)
Supplement: Supplementary file 6 [file DataSheet1.pdf]

Supplemental Information for

Auxin-inducible degron system reveals temporal-spatial roles of HSF-1 and its transcriptional program in lifespan assurance

Allison C. Morphis[1,2], Stacey L. Edwards[1,2], Purevsuren Erdenebat[1], Lalit Kumar[1], and Jian Li[1*]

[1] Aging and Metabolism Research Program, Oklahoma Medical Research Foundation,

Oklahoma City, OK, USA

[2]These authors contribute equally.

Figure S1. Fecundity measurement upon HSF-1 depletion as young adults.

(A) Fecundity of HSF-1 AID worms during the self-reproductive period. Experiments were done with mock treatment (no HSF-1 depletion) in three biological replicates (n>=10 in each replicate). Data are represented as mean ± SEM (n>=10).

(B) Fecundity of animals with HSF-1 depleted in the germline or in the soma at young adult stage. Animals were treated with auxin to induce HSF-1 depletion or mock treated with ethanol as the control. Data are represented as mean ± standard deviation (n>=12).

Figure S2. Temporal requirement for HSF-1 in long-lived animals.

(A&B) Representative live animal images of HSF-1 AID worms in the *glp-1(2141)* (A) and *fem-3*(q20) (B) background on Day 4. Animals were treated with auxin for 2 h to induce HSF-1 depletion in the soma or mock treated as the control. N: neuronal ring; I: intestinal nucleus; H: hypodermic nucleus; A: auto-fluorescence in puncta (not HSF-1::GFP).

(C) Lifespan analysis at 20°C upon pan-somatic depletion of HSF-1 by AID in the *daf-2 (e1370)* background. The control strain, JTL618 (*daf-2; eft-3p::tir1*) was mock treated with ethanol (EtOH) or treated with auxin since Day 1 of adulthood. The HSF-1 AID model, JTL641 (*daf-2; eft-3p::tir1; hsf-1::degron)* was mock treated with ethanol (EtOH) since Day 1 of adulthood or transferred from EtOH to auxin plates at indicated time to initiate HSF-1 depletion.

(D) Lifespan analysis at 20°C upon pan-somatic depletion of HSF-1 by AID in the *daf-2 (e1370)* background as in panel C except that animals were fed with carbenicillin treated OP50 bacteria and HSF-1 depletion was initiated at different time points.

Figure S3. Transcriptional program of HSF-1 in the soma of young adults.

(A&B) Histograms showing the number of differentially expressed (DE) genes caused by auxin treatment (A) or due to degron insertion to endogenous *hsf-1* (B).

(C) Heatmap of mRNA fold change upon auxin treatment in CA1200 (*eft-3p::tir1*) that expresses TIR1 in the soma and CA1199 (*sun-1p::tir1*) that expresses TIR1 in the germline (germ). Whole

animal RNA-seq analyses were done in three biological replicates in young adults treated with auxin for 2 h, 8 h and 24 h. The mRNA fold change (auxin/ethanol control) is shown. Differentially expressed genes (FDR: 0.05) at any time point were included and grouped by kmeans clustering (n=6).

(D) Scatter plot showing the two methods either by comparing the experimental strain JTL611 (*eft-3p::tir1; hsf-1::degron eft-3p::tir1; hsf-1::degron)* to control strain CA1200 (*eft-3p::tir1)* when both treated by auxin, or by comparing the JTL611 that is mock treated to auxin treated, resolved similar expression changes. DE genes at 24 h HSF-1 depletion were shown.

(E) Venn diagram showing the overlap of down-regulated genes upon HSF-1 depletion in the soma (24 h in JTL611) and genes with HSF-1 binding at the promoters in the soma (either specifically enriched in the soma or shared between the soma and the germline). P-value: Fisher's exact test.

(F) The gene network directly activated by HSF-1 in the somatic cells in the wild-type background. Genes included are those with HSF-1 binding peaks at the promoters and significantly decreased expression upon HSF-1 depletion from the soma for 8 h or 24 h on Day 1 of adulthood. The protein-protein interaction network was retrieved from STRING database and grouped by kmeans clustering (n=2). The node color represents the cluster which the gene belongs to. The color saturation of edges represents the confidence score of a functional interaction. Genes with names in black encode chaperones or co-chaperones, and genes with names in red are those with other functions.

(G&H) Representative images showing immunofluorescence of ubiquitinylated proteins in the control group (G) or upon HSF-1 depletion (H) since Day 1 of adulthood from the soma in *glp-1(e2141)* for 48 h. Animals were stained by anti-REC-8 (enriched in nuclei) to control antibody accessibility.

(I&J) Histograms showing mRNA levels (from RNA-seq) of candidates of HSF-1 directly regulated genes in *fem-3(q20)* and *glp-1(e2141)* upon HSF-1 depletion for 24 h. The 'fold decrease' of

mRNA levels upon HSF-1 depletion <log2 (control/HSF-1 depletion)> (I) and the absolute mRNA levels in CPM (counts per million) (J) are shown.

Figure S4. AID models for depletion of HSF-1 in specific somatic tissues

(A-D) Representative live animal images of HSF-1 AID worms in the *glp-1(2141)* background on Day 1. Animals expressing TIR1 in the hypodermis (A), body wall muscle (B), intestine (C) or neural system (D) were treated with auxin for 2 h to induce HSF-1 depletion or mock treated as the control. For the AID models of the hypodermis, body wall muscle and intestine (A-C), only the head region is shown as the tissue-specificity of HSF-1 depletion is the same for the rest of body. For the AID model of the nervous system (D), auxin treatment seemed to impair HSF-1 levels in the first pairs of intestinal nuclei from the head as we had difficulty observing HSF-1::GFP signal in those nuclei, but not the nuclei from the center to the tail. To avoid the interference of auto-fluorescence in the intestine, maximal projection of only a subset of z-stack is shown, and therefore HSF-1::GFP is only visible from the nuclei that fall onto the focal planes. N: neuronal ring; I: intestinal nucleus; H: hypodermic nucleus; M: body wall muscle nucleus.

(E) Representative live animal images of worms with pan-somatic expression of degron::GFP and neural expression of TIR1. Animals were treated with auxin for 2 h to induce GFP depletion or mock treated as the control. N: neuronal ring; I: intestinal cell; A: auto-fluorescence in puncta (not degron::GFP).

Figure S5. Different tissue requirements for HSF-1 in larval development and lifespan assurance

(A-C) Size tracking of developing larvae with continuous HSF-1 depletion in the intestine (A), neurons (B) and muscle (C) initiated at egg lay. Experiments were done in the wild-type background at 20°C. Data are represented as mean ± standard deviation (n>=12). P<0.0001 (control vs. HSF-1 depletion, two-way ANOVA) for all three tissues.

(D) Representative image showing the molting defect of one worm that had HSF-1 depleted in the hypodermis since egg lay. The animal died during molting when the cuticle was separated from the head. Scale bar: 50μm.

(E) Mobility of developing larvae measured as body length per second with continuous HSF-1 depletion in the neurons initiated at egg lay. Experiments were done in the wild-type background at 20°C. Data are represented as mean ± standard deviation (n>=12). $P<0.0001$ (control vs. HSF-1 depletion, two-way ANOVA).

(F) Representative image showing internal hatching ('bagging') in a Day 2 adult that had HSF-1 depleted in the muscle since egg lay. Arrow head indicates a larva that was hatched inside the mother. Scale bar: 50 μm.
